# Commensal and Pathogenic Bacteria Indirectly Induce IL-22 but Not IFNγ Production From Human Colonic ILC3s via Multiple Mechanisms

**DOI:** 10.3389/fimmu.2019.00649

**Published:** 2019-03-29

**Authors:** Moriah J. Castleman, Stephanie M. Dillon, Christine M. Purba, Andrew C. Cogswell, Jon J. Kibbie, Martin D. McCarter, Mario L. Santiago, Edward Barker, Cara C. Wilson

**Affiliations:** ^1^Division of Infectious Disease, Department of Medicine, University of Colorado Anschutz Medical, Aurora, CO, United States; ^2^Department of Microbial Pathogens and Immunity, Rush University Medical Center, Chicago, IL, United States; ^3^Department of Surgery, University of Colorado Anschutz Medical, Aurora, CO, United States

**Keywords:** human, innate lymphoid cells, commensal bacteria, colonic mucosa, NKp44, myeloid dendritic cells, IL-23, IL-1β

## Abstract

Innate lymphoid cells (ILCs) are a diverse family of cells that play critical roles in mucosal immunity. One subset of the ILC family, Group 3 ILCs (ILC3s), has been shown to aid in gut homeostasis through the production of IL-22. IL-22 promotes gut homeostasis through its functional effect on the epithelial barrier. When gut epithelial barrier integrity is compromised, such as in Human Immunodeficiency Virus (HIV) infection and inflammatory bowel disease (IBD), microbes from the gut lumen translocate into the lamina propria, inducing a multitude of potentially pathogenic immune responses. In murine models of bacterial infection, there is evidence that bacteria can induce pro-inflammatory IFNγ production in ILC3s. However, the impact of diverse translocating bacteria, particularly commensal bacteria, in dictating IFNγ versus IL-22 production by human gut ILC3s remains unclear. Here, we utilized an *in vitro* human lamina propria mononuclear cell (LPMC) model to evaluate ILC3 cytokine production in response to a panel of enteric Gram-positive and Gram-negative commensal and pathogenic bacteria and determined potential mechanisms by which these cytokine responses were induced. The percentages of IL-22-producing ILC3s, but not IFNγ-producing ILC3s, were significantly increased after LPMC exposure to both Gram-positive and Gram-negative commensal or pathogenic bacterial stimuli. Stimulation of IL-22 production from ILC3s was not through direct recognition of bacterial antigen by ILC3s, but rather required the help of accessory cells within the LPMC population. CD11c+ myeloid dendritic cells generated IL-23 and IL-1β in response to enteric bacteria and contributed to ILC3 production of IL-22. Furthermore, ligation of the natural cytotoxicity receptor NKp44 on ILC3s in response to bacteria stimulation also significantly increased the percentage of IL-22-producing ILC3s. Overall, these data demonstrate that human gut microbiota, including commensal bacteria, indirectly modulate colonic ILC3 function to induce IL-22, but additional signals are likely required to induce IFNγ production by colonic ILC3s in the setting of inflammation and microbial translocation.

## Introduction

Innate Lymphoid Cells (ILCs) descend from the lymphoid lineage, but unlike T cells and B cells lack rearranged antigen receptors (1). Rather than responding to antigen priming, ILCs respond in an antigen-independent manner through cytokine stimulation to produce effector cytokines (2) or through engagement of germline encoded receptor stimulation (3–5). ILCs are categorized into three groups based on the expression of specific master transcription factors and effector function (6). Group 1 ILCs (ILC1, NK cells) express T-bet and produce the pro-inflammatory cytokine IFNγ in response to IL-12 and IL-18; Group 2 ILCs (ILC2s) express GATA3 and produce IL-4, IL-13, and IL-5 in response to IL-25 and IL-33; and Group 3 ILCs (ILC3s) express RORγt and AHR and typically produce IL-22, GM-CSF, IL-17, and LT-α1β2 in response to IL-23 and IL-1β (6). ILCs are constitutively found in the gastrointestinal tract (7). ILC3s are of particular importance to gut mucosal immunity (7–9) via the secretion of effector cytokines that act directly on epithelial cells (10). Epithelial cells express the IL-22 receptor (IL-22R) (11) and IL-22 production by ILC3s promotes epithelium proliferation, survival, mucus production, upregulation of fucosylation, and in some studies increased gene expression of antimicrobial peptides (12–16). To date, the majority of studies characterizing gut ILC3 function have utilized murine models. Examination of human ILC3 function have primarily focused on tonsil tissue and demonstrated that the mechanisms contributing to IL-22 production include stimulation by the cytokines IL-23 and IL-1β (3, 17, 18) with synergistic enhancement of IL-22 production observed in the presence of the natural cytotoxicity receptor NKp44 engagement (3). Few studies have directly investigated factors driving IL-22 production by human gut ILC3s, although one study observed a requirement for IL-23, IL-1β, and IL-7, with synergy again being induced in the presence of NKp44 signaling (3).

Epithelial barrier damage and loss of function in gut-associated diseased states have correlated with alterations in ILC frequency and function. In Inflammatory Bowel Disease (IBD), patients with Crohn's disease have a loss of colonic or ileum IL-22-producing ILCs (including ILC3s) (18, 19) and an increase in IFNγ/IL-17A-producing ILCs (20–22). In Human Immunodeficiency Virus (HIV) infection, loss of colonic IL-22-producing ILCs has been reported (23). Similarly, reduced frequencies of IL-22/IL-17-producing ILCs during Simian Immunodeficiency Virus (SIV) infection (the non-human primate model of HIV) were noted (24–28). Furthermore, we and others have reported increased frequencies of IFNγ-producing ILCs both in people living with HIV (PLWH) who were not receiving anti-retroviral therapy (ART) (29) and during SIV infection (27). Since IFNγ alters epithelial tight junctions and upregulates epithelial cell expression of TNFα receptor which results in further epithelial cell damage (30, 31), increased frequencies of IFNγ-producing ILC3s may be an additional contributor to epithelial barrier breakdown. When the epithelial barrier is compromised, translocation of gut-associated bacteria into the lamina propria (LP) exposes immune cells to bacteria of different species or magnitude than what these cells typically encounter in the healthy human gut (32, 33). We previously demonstrated that increased frequencies of colonic IFNγ-producing ILCs in PLWH correlated with alterations in mucosa-associated bacterial communities (dysbiosis), specifically with increased relative abundance of Gram-negative commensal *Prevotella* species (29). Understanding the bacteria-specific cytokine responses of ILC3s and the mechanisms by which protective or deleterious cytokines are produced are critical to determining the effect of ILC3s on gut homeostasis, not only for their role in enteric bacterial immunity, but also for their role in influencing epithelial cell function in disease states.

Murine studies highlighted a complex role for gut microbiota in ILC subset development and functional production of IL-22 (13, 34, 35). IL-22 production by ILC3s protected against an enteric pathogen *Citrobacter rodentium* (34, 36, 37), and prevented systemic dissemination of the commensal *Alcaligenes* species in mice (15). Fucosylation of epithelial cells induced by ILC3 production of IL-22 contributed to host defense against murine *S. typhimurium* infection (38). Furthermore, murine ILC3s negatively regulated microbe-specific T cells in the gut to limit pathological responses to commensal bacteria (39, 40). While these studies support a homeostatic role for ILC3s in microbiota-associated gut responses in mice, gut inflammatory ILC3s in response to bacteria have been reported. ILC3s produced IFNγ in response to infection with *Salmonella typhimurium* (41) and IFNγ/IL-17 in response to infection with the *Helicobacter hepaticus* (42). Furthermore, ILC3-associated IFNγ/IL-17 production in response to *H. hepaticus* was linked to the development of colitis (42) highlighting a potentially deleterious role of ILC3 cytokine production. *In vitro* exposure of human ILC3s have also suggested a plasticity in cytokine production with the capacity to produce IFNγ or IL-22 dependent on the cytokine milieu (18, 21). These observations raise the possibility that human gut ILC3s may also have the capacity to produce IL-22 or IFNγ in response to exposure to different types of bacteria.

In this study, we hypothesized that pathogenic enteric bacteria would induce pro-inflammatory cytokine production (IFNγ) from human lamina propria ILC3s, whereas commensal bacteria would primarily elicit protective (IL-22) cytokine production. To address this, we utilized an *in vitro* human colonic mononuclear cell model (43, 44) to investigate ILC3 cytokine profiles induced in response to a panel of whole Gram-negative and Gram-positive, commensal and pathogenic bacteria and the mechanisms driving these responses. Overall, our observations provide insight into the ILC3 role in enteric bacteria immunity and their contribution to the inflammatory environment in disease states where microbes translocate through a compromised epithelial barrier.

## Materials and Methods

### Human Tissue Samples

Human colonic tissue samples were acquired from patients undergoing elective abdominal surgery at the University of Colorado Hospital and are categorized as discarded tissue from macroscopically normal sites. Samples from patients that underwent chemotherapy or radiation within 8 weeks of tissue collection were not included in the study. Other criteria for tissue exclusion include those with Inflammatory Bowel Disease, HIV infection or treatment with immunosuppressive drugs. Intraepithelial mononuclear cells (IEMC) or lamina propria mononuclear cells (LPMC) were isolated from tissue samples as previously described (43, 44) and stored in liquid nitrogen until use. Human tonsillar tissue samples were acquired from pediatric patients from Colorado Children's Hospital. Tonsillar mononuclear cells (TMCs) were isolated as previously described (45). All patients undergoing surgery signed a release to allow unrestricted use of discarded tissue and protected patient information was de-identified to the laboratory investigators. This research was reviewed by the Colorado Multiple Institutional Review Board (COMIRB) at the University of Colorado Anschutz Medical Campus and was granted exempt research status.

### Preparation of Bacterial Stocks

Growth of anaerobic bacteria was performed using a BD GasPak EZ Anaerobe Pouch System according to manufacturer's instructions (BD Diagnostics, Franklin Lakes, NJ). *Prevotella stercorea* (DSM No. 18206, Braunschweig, Germany) was grown in liquid chopped meat broth (Hardy Diagnostics, Santa Maria, CA) supplemented with 1% Trace Minerals (ATCC), 1% Vitamin Supplements (ATCC), 0.05% Tween80, 29.7 mM acetic acid, 8.1 mM propionic acid and 4.4 mM butyric acid (Sigma-Aldrich) under anaerobic conditions at 37°C for 5–7 days. *Ruminococcus bromii* (ATCC# 27255) was grown in liquid chopped meat broth (Hardy Diagnostics) under anaerobic conditions at 37°C for 1–2 days. The long term stock of *Bifidobacterium longum* subsp *infantis* (ATCC 15697) was grown in liquid chopped meat broth (Hardy Diagnostics) under anaerobic conditions at 37°C for 2–3 days and the working stock was grown on Brucella plates (Teknova, Hollister, CA) under anaerobic conditions at 37°C for 2–3 days. *Acinetobacter junii* (ATCC 17908) was grown using Nutrient Agar plates (Edge Biologicals, Memphis, TN) under aerobic conditions at 26°C for 1–2 days. *Salmonella typhimurium* (ATCC 35986) was grown on LB agar plates (Sigma-Aldrich) under aerobic conditions at 37°C for 1–2 days. Long term stocks of all bacteria were prepared using 10% glycerol and single-use working stocks were prepared using DPBS. All stocks were stored at −80°C and bacterial cell counts were determined using the BD Cell Viability Kit (BD Bioscience).

### *In vitro* Stimulation of LPMCs With Whole Bacteria

For the *in vitro* stimulations, human colonic LPMCs were thawed as previously described (43, 44) and cultured in RPMI with 10% human AB serum (Gemini Bioproducts, West Sacramento, CA), 1% Penicillin/Streptomycin/Glutamine (Life Technologies, Grand Island, NY), and 500 μg/ml Zosyn (Piperacillin and Tazobactam, Wyeth, Madison, NY) at a concentration of 1.0 × 10^6^ million cells per mL in a 48 well plate. LPMCs were exposed to a panel of Gram-positive and Gram- negative bacteria detailed in Supplemental Table 1 including mucosa-associated colonic bacteria previously shown to be increased or decreased in relative abundance during HIV-1 infection (46, 47): Gram-negative *Prevotella stercorea* and *Acinetobacter junii* (increased) and Gram-positive *Ruminococcus bromii* (decreased), as well as the Gram-positive probiotic *Bifidobacterium infantis* and the Gram-negative pathogen *Salmonella typhimurium*. Broad spectrum antibiotics including Penicillin, Streptomycin, Piperacillin, and Tazobactam were present throughout the time in culture to prevent bacterial overgrowth.

For assays examining ILC3 responses, whole bacteria were added to cell cultures at a ratio of 2.5 bacteria to 1 LPMC and incubated for 16 h at 37°C + 5% CO_2_, followed by the addition of Golgi Plug Transport Inhibitor (BD Bioscience) for 4 h. Cells were then collected for flow cytometry as described below.

For blocking experiments: human colonic LPMCs were first exposed to blocking antibodies targeting IL-23 p19, IL-1β (R & D Systems, Minneapolis, MN) or IL-7 (Biolegend) at 5 ug/mL or NKp44 (Biolegend) at 10 ug/mL for 30 min followed by incubation with *A. junii* as described above. Cells were then collected for flow cytometry as described below. The concentration of blocking antibodies was optimized for use by measurement of IL-22 + ILC3s or IL-22 + Lineage negative cells (for IL-7) in response to recombinant cytokine stimulation (50 ng/mL of IL-23, IL-1β, or IL-7) or bead ligation (for NKp44) with a dose curve of blocking antibody treatment (Supplemental Figure 1). The addition of recombinant IL-7 prevented the identification of ILC3s (defined as CD127+ which is IL-7Rα) and instead gating for IL-22 was determined on lineage- cells. Antibodies and controls used to block are listed in Supplemental Table 2.

For depletion experiments: CD11c+ cells (mDC) or CD3+ (T cells) were depleted from LPMCs using the EasySep PE Positive Selection Kit according to manufacturer's instructions (StemCell Technologies, Vancouver, Canada) and the antibody PE-CD11c (Biolegend, San Diego, CA) or PE-CD3 (Tonbo, San Diego, CA) followed by incubation with *A. junii* as described above. Greater than 90.25% of CD11c+ mDCs were depleted from total LPMCs. Greater than 91.92% of CD3+ T cells were depleted from total LPMCs. Cells were then collected for flow cytometry as described below.

For the measurement of secreted cytokines, LPMCS were plated at a concentration of 2.0 × 10^6^ million cells per mL in a 96 well plate and exposed to whole bacteria *R. bromii* or *A. junii* at a ratio of 2.5 bacteria to 1 LPMC and incubated for 24 h at 37°C + 5% CO_2_. Supernatant was collected and saved at −20°C until use. IL-23, IL-1β, and IL-7 were measured in the supernatant using the U-PLEX Assay according to manufacturer's instructions and quantified on the QuickPlex SQ 120 Instrument (Mesoscale Discovery, Rockville, MD).

For assays examining antigen presenting cell responses (mDC, B cell, and Macrophages), whole bacteria were added to cell cultures at 2.5 bacteria to 1 LPMC and incubated for 4 h at 37°C + 5% CO_2_, followed by the addition of Golgi Plug Transport Inhibitor (BD Bioscience) for 16 h. Cells were then collected for flow cytometry as described below.

### *In vitro* Stimulation of LPMCs With Bacterial Cell Surface Components

Human colonic LPMCs were exposed to either 1 μg/mL of the TLR2 ligand, lipoteichoic acid (LTA) from *B. subtilis* (InvivoGen, San Diego, CA), or the TLR4 ligand, lipopolysaccharide (LPS) from *E. coli* (InvivoGen) for 16 h at 37°C + 5% CO_2_, followed by the addition of Golgi Plug Transport Inhibitor (BD Bioscience) for 4 h. Cells were then collected for flow cytometry as described below.

### *In vitro* Stimulation of LPMCs With Recombinant Cytokines or NKp44 Activation

Human colonic LPMCs were exposed to 50 ng/mL IL-23 or IL-1β (R & D Systems), or the combination of both, or IL-2 or IL-7 (Tonbo) for 16 h at 37°C + 5% CO_2_ followed by the addition of Golgi Plug Transport Inhibitor (BD Bioscience) for 4 h. For NKp44 activation experiments; 20 ug/mL of anti-NKp44-biotin (clone P44-8, Biolegend) was combined with Anti-Biotin MACSiBead Particles (Miltenyi Biotec) according to manufacturer's instructions. The beads were then added to LPMCs at a ratio of 5 beads to 1 LPMC for 16 h at 37°C + 5% CO_2_ followed by the addition of Golgi Plug Transport Inhibitor (BD Bioscience) for 4 h. Cells were then collected for flow cytometry as described below.

### *In vitro* Stimulation of Tonsil Mononuclear Cells

Human tonsil mononuclear cells (TMCs) were cultured in RPMI with 10% human AB serum (Gemini Bioproducts), 1% penicillin/streptomycin/glutamine (Life Technologies), and 500 μg/ml Zosyn (Wyeth) at a concentration of 1.0 × 10^6^ million cells per mL in a 48 well plate. TMCs were exposed to whole bacteria added to cell cultures at a ratio of 2.5 bacteria to 1 LPMC, or to the combination of 50 ng/mL IL-23 and IL-1β, or NKp44 activation beads at a ratio of 5 beads to 1 TMC and incubated for 16 h at 37°C + 5% CO_2_, followed by the addition of Golgi Plug Transport Inhibitor (BD Bioscience) for 4 h. Cells were then collected for flow cytometry as described below.

### Flow Cytometry Protocol for Surface and Intracellular Staining

Using flow cytometry, viable CD45+ single cell lymphocytes were identified followed by identification of ILC3s as follows: Lineage-CD127+CD117+. The lineage negative cocktail comprised antibodies targeting CD3, CD20, CD13, CD123, CD303, CD34, FCεR1α, CD11c, and CRTH2. All data were acquired on an LSRII flow cytometer (BD Biosciences). Routine quality control using the Cytometer Setup and Tracking feature within the BD FACSDiva software version 6.1.2 (BD Biosciences) was performed daily. All antibodies and clones used for staining are listed in Supplemental Table 3.

For *ex vivo* phenotyping of ILC3s: IEMCs or LPMCs were thawed and surface stained to identify ILC subsets for expression of NKp44, CD56, CCR6, TLR2, TLR4, and TLR5 and intranuclear stained for the transcription factors RORγt, AHR, T-bet and EOMES using the Foxp3/Transcription Factor buffer set according to manufacturer's instructions (Thermo Fisher Scientific, Frederick, MD).

For *in vitro* culture examination of ILC3s; LPMCs were collected after stimulations described above and surface stained to identify ILC3s and subsets (NKp44) followed by intracellular staining for cytokines IL-22, IFNγ, and IL-17 using Fix and Perm Cell fixation and permeabilization buffer set according to manufacturer's instructions (Thermo Fisher Scientific, Frederick, MD).

For *in vitro* culture examination of antigen presenting cells; cells were collected after stimulation and surface stained to identify mDCs (CD45+ Viable Myeloid CD3- CD19- HLA-DR+ CD11c+), B cells (CD45+ Viable Lymphocyte CD3- CD19+) or Macrophages (CD45+ Viable Myeloid CD3- CD19- HLA-DR+ CD11c-) [as defined by Smith et al. and Bain and Mowat (48–50)] followed by intracellular staining for cytokines IL-12/IL-23 p40, IL-23p19, and IL-1β using Fix and Perm Cell fixation and permeabilization reagents according to manufacturer's instructions (Thermo Fisher Scientific). Only cells that expressed both subunits of the cytokine IL-23 (IL-12/IL-23p40 and IL-23p19) were considered to be IL-23+ cells.

### *In vitro* Stimulation of Purified ILC3s

ILC3s were isolated from colonic LPMCs and on average purified to 91.79% purity (Supplemental Figure 2) by sorting using the MoFlo Astrios EQ (Beckman Coulter, Indianapolis, IN). ILC3s were sorted from Viable CD45+ single cell lymphocytes that were Lineage-CD127+CD117+ as described above. Purified ILC3s were then exposed to either 50 ng/mL IL-23 and IL-1β (R & D Systems) or whole bacteria at a ratio of 1 bacteria to 1 ILC3 and incubated for 24 h at 37°C + 5% CO_2_. Supernatant was collected and saved at −20°C until use. Secreted IL-22 was measured in the supernatant using the IL-22 U-PLEX Assay according to manufacturer's instructions and quantified on the QuickPlex SQ 120 Instrument (Mesoscale Discovery, Rockville, MD).

### Data Analysis

Each patient who provided a tissue specimen for research is considered a single sample for data analysis, and figure legends indicate how many samples were examined for each assay using the following terminology: *N* = number of patients samples. All flow cytometer data analysis was done using FlowJo v10.0. All statistical analysis and graphing were performed using GraphPad Prism v6.00 for Windows (GraphPad Software, La Jolla California). Paired *t*-test was used to determine statistical differences between conditions as indicated in figure legend. Data sets without a minimum number of 25 ILC3 events captured using flow cytometry were excluded from analysis.

## Results

### ILC3s Are Phenotypically Similar in the Intraepithelial and Lamina Propria Layers of the Human Colon

**Figure 1:**
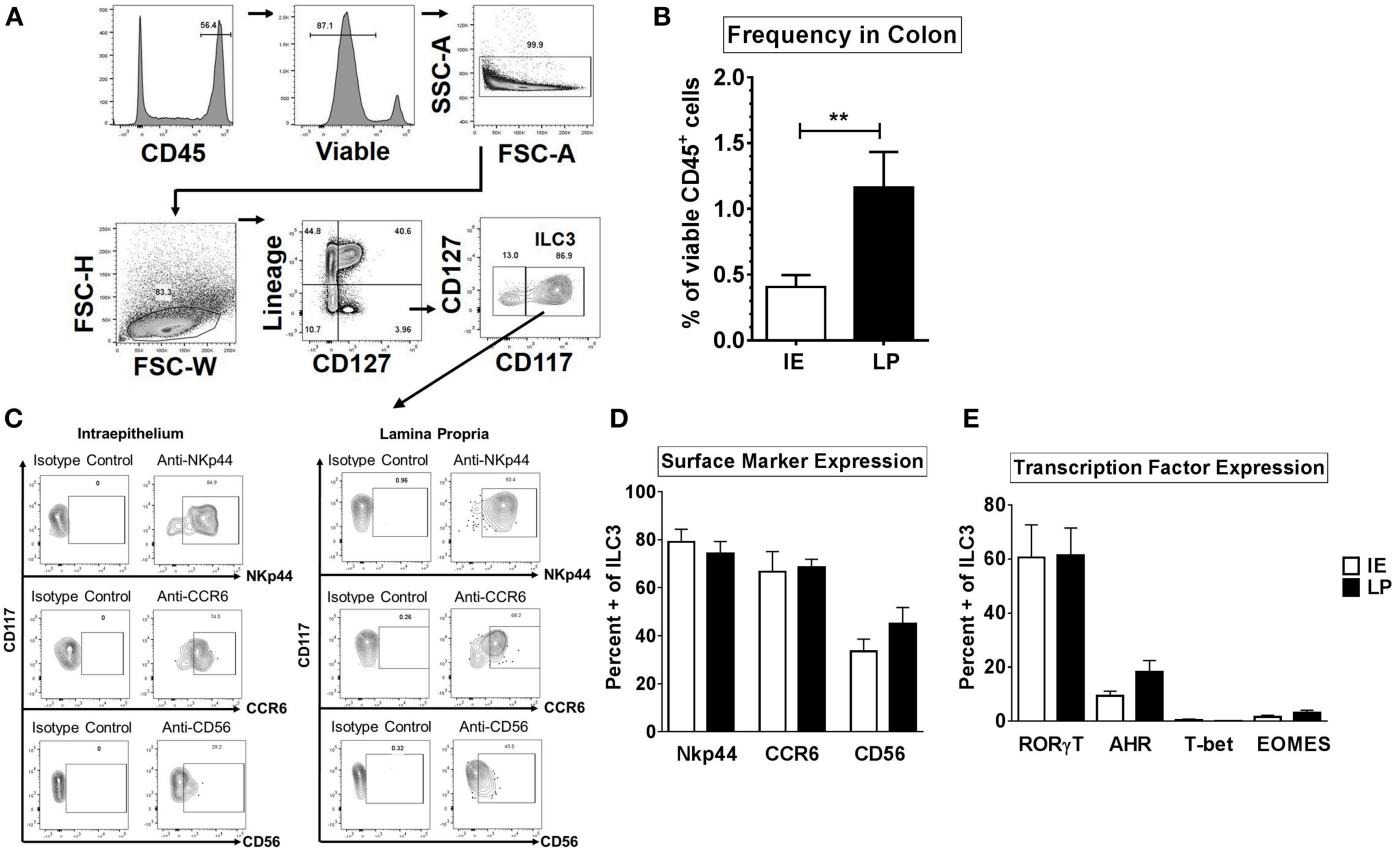
ILC3s are phenotypically similar between the intraepithelial and lamina propria layers of the human colon. **(A)** Representative flow cytometry gating strategy to identify ILC3s in human colonic tissue. **(B)** Frequencies of ILC3s in the colon in the intraepithelial (IE) and lamina propria (LP) layer as a percent of viable CD45+ cells. IE: *N* = 6, LP: *N* = 6. **(C)** Representative flow plots gated on ILC3s for the expression of surface markers NKp44, CD56, or CCR6. **(D)** Percentages of ILC3s expressing the surface markers NKp44, CD56, or CCR6 *ex vivo*. IE: *N* = 6, LP: *N* = 6. **(E)** Percentages of ILC3s expressing the transcription factors RORγt, AHR, T-bet, or EOMES *ex vivo*. IE: *N* = 7, LP: *N* = 7. Bars are mean + S.E.M. Statistical analysis performed was paired t test. ^**^p < 0.01.

Human ILC1 subsets were previously shown to be phenotypically different between the colonic layers (22). We therefore sought to determine if ILC3s that reside in the intraepithelial layer (IE) of the human colon (closer to the intestinal lumen where gut bacteria reside) are phenotypically different to ILC3s that reside within the lamina propria layer (LP). The frequency and phenotype of ILC3 in these locations were determined with ILC3s in colonic human tissue identified as CD45+ viable Lineage- CD127+ CD117+ (Figure 1A). ILC3s were more frequent as a fraction of CD45+ cells in the LP (1.16% ± 0.27) compared to the IE layer (0.40% ± 0.09) (Figure 1B).

NKp44, CD56, and CCR6, have previously been utilized to identify subsets of ILC3s (17, 24, 29, 41) therefore expression of these markers was next evaluated on colonic ILC3s. The majority of ILC3s expressed NKp44 or CCR6 in both layers (NKp44: IE: 79.13% ± 5.25, LP: 74.21% ± 5.04; CCR6: IE: 66.68% ± 8.40, LP: 68.61% ± 3.27) (Figures 1C,D). On average, less than half of LP and IE ILC3s expressed CD56 (LP: 45.08% ± 6.68; IE: 33.58% ± 5.04) (Figures 1C,D).

As expected (51), the majority of ILC3s expressed the master transcription factor RORγt (IE: 60.60% ± 12.07, LP: 61.42% ± 10.10) and frequencies of RORγt + ILC3s as a percent of viable CD45 + lymphocytes were not significantly different between the IE and LP layers (Figure 1E and Supplemental Figure 3). Expression of AHR in ILC3s was lower than that of RORγt (Figure 1E and Supplemental Figure 3) but similar between tissue layers (LP: 18.23% ± 4.24, IE: 9.35% ± 1.77). Of the AHR-expressing ILC3s, the majority also co-expressed RORγt (LP: 76.77% ± 8.84, IE: 82.44% ± 9.74). Less than 1% of ILC3s expressed T-bet in both layers of the colon (LP: 0.15% ± 0.06, IE: 0.67% ± 0.49) (Figure 1E and Supplemental Figure 3). Low frequencies of EOMES expressing ILC3s were also quantified in both layers (LP: 1.56% ± 0.62, IE: 3.16% ± 0.91). Overall, of the markers examined, ILC3s were phenotypically similar between the intraepithelial and lamina propria layer in the normal human colon.

### Enteric Bacteria Stimulate Production of IL-22 but Not IFNγ From ILC3s When Exposed to Total LPMCs

**Figure 2:**
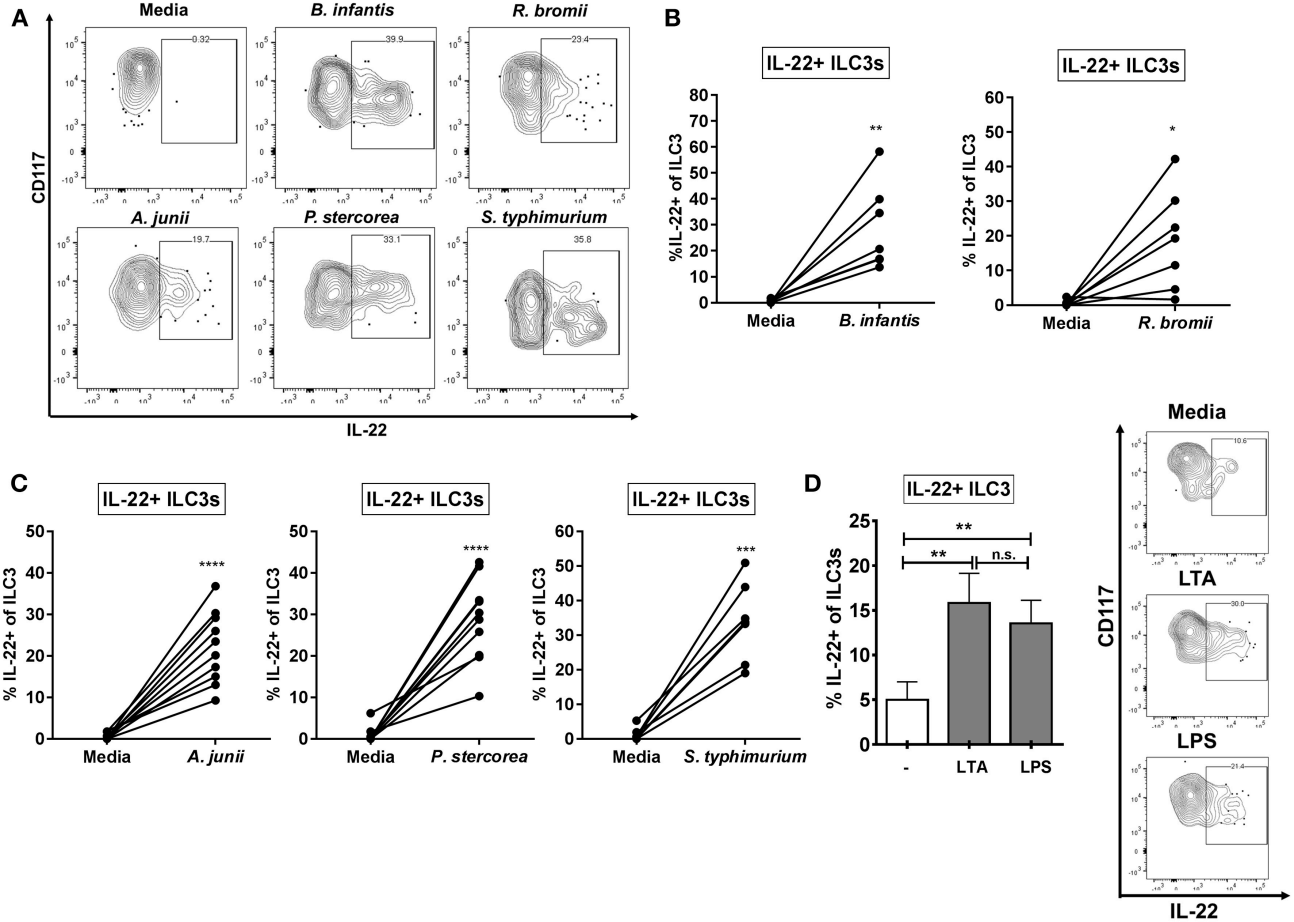
ILC3s produce IL-22 in response to Gram-positive and Gram-negative enteric bacteria. **(A)** Representative flow cytometry demonstrating cytokine staining for IL-22 gated on ILC3s after LPMC exposure to enteric bacteria *in vitro*. **(B,C)** Percentages of IL-22+ ILC3s after LPMC exposure to enteric bacteria or no bacterial control. *N* = 6–10. **(D)** Percentages of IL-22+ ILC3s after LPMC exposure to lipoteichoic acid (LTA) or lipopolysaccharide (LPS) or no stimulation control. Representative flow cytometry demonstrating cytokine staining for IL-22 gated on ILC3s after LPMC exposure to bacterial cell surface components. *N* = 6. Bars are mean + S.E.M. Statistical analysis performed was paired t test as indicated. ^*^p < 0.05, ^**^p < 0.01, ^***^p < 0.001, ^****^p < 0.0001. n.s., not significant.

Mimicking the state where the epithelial barrier is damaged and LP immune cells are exposed to bacteria from the colonic lumen, we utilized an *in vitro* model of lamina propria mononuclear cells (LPMCs) to investigate the ILC3 response to whole enteric bacteria. LPMCs were exposed to a panel of whole bacteria representing Gram-positive and Gram-negative commensal bacteria reported to be altered in various diseases associated with epithelial barrier damage (Supplemental Table 1). This panel included commensal Gram-positive *Ruminococcus bromii* (Rb) which is decreased in relative abundance in colonic mucosa of people living with HIV (PLWH) and Gram-negative *Acinetobacter junii* (Aj) and *Prevotella stercorea* (Ps), which were increased in relative abundance (46, 47). The enteric gram-negative pathogen *Salmonella typhimurium* (St) which may contribute to the onset of IBD symptoms (52) and to which PLWH are at an increased risk of acquiring *S. typhiumurium*-bacteremia (53, 54) as well as the probiotic Gram-positive *Bifidobacterium longum* sp *infantis* (Bi), which is used therapeutically to reestablish a protective microbiome (55, 56) were also included. Bacterial experiments were performed in the presence of broad spectrum antibiotics in order to inhibit bacterial overgrowth. All bacteria tested, irrespective of gram-staining and commensal or pathogenic nature, induced production of IL-22 in a fraction of ILC3s relative to the no stimulation condition (Bi: 30.0% ± 7.13, Rb: 18.76% ± 6.43, Aj: 21.61% ± 3.02, Ps: 28.58% ± 3.54, St: 32.21% ± 4.65) (Figures 2A–C). Of the bacteria tested, none induced a significant increase in IL-17A+ ILC3s above the no stimuli control (data not shown: less than 0.80% IL-17+ ILC3).

To determine if bacterial surface components were important in driving the ILC3 IL-22 response to whole bacteria, LPMCs were exposed to Gram-positive and Gram-negative bacterial cell surface components (LTA and LPS, respectively). The percentage of IL-22+ ILC3s was similarly increased in response to both bacterial surface antigens (LTA: 17.98% ± 3.09, LPS: 15.18% ± 2.44) (Figure 2D).

ILC3s that produced IFNγ were associated with inflammatory responses in murine infection with bacteria (41) or bacteria-driven murine colitis (42). We next measured the frequencies of IFNγ-producing LP ILC3s in response to whole bacteria. Low frequencies of IFNγ expressing ILC3s were observed in response to exposure of LPMC to each bacteria (Bi: 0.63% ± 0.50, Rb: 0.06% ± 0.04, Aj: 1.51% ± 0.81, Ps: 0.50% ± 0.35, St: 1.77% ± 0.68), however no significant increase above the no stimuli control was detected (Supplemental Figure 4). Furthermore, IFNγ+ ILC3s were not induced by exposure to LTA and LPS (data not shown). Overall, these data indicate that the bacteria tested did not drive the production of IFNγ by ILC3s in our *in vitro* culture system.

### Purified ILC3s Do Not Produce IL-22 in Response to Enteric Bacteria

**Figure 3:**
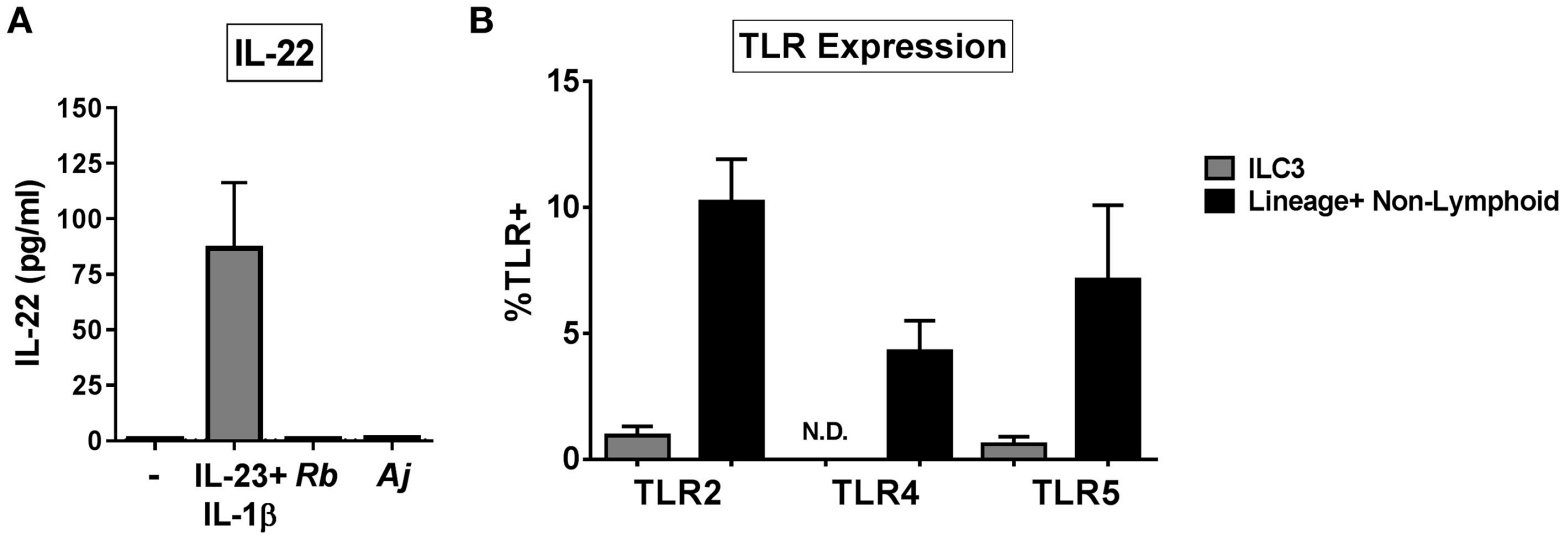
ILC3s do not respond directly to bacteria by producing IL-22. **(A)** Quantification of IL-22 (pg/mL) in the supernatant of purified ILC3s exposed to recombinant IL-23+ IL-1β (50 ng/mL) or *R. bromii* (Rb) or *A. junii* (Aj) *in vitro* at a ratio of 1 ILC3 to 1 bacterium or no stimulation control. *N* = 3. **(B)** Percentages of LPMCs stained *ex vivo* for TLR2, TLR4, or TLR5 expression gated on ILC3s or Lineage positive non-lymphoid cells. *N* = 6. N.D., not detected. Bars are mean + S.E.M.

To assess if ILC3s produced IL-22 in direct response to bacteria, ILC3s were purified from the LP (Supplemental Figure 2) and exposed *in vitro* to either Gram-positive (*R. bromii)*, or Gram-negative (*A. junii)* bacteria or to recombinant IL-23 and IL-1β. After 24 h, the levels of secreted IL-22 were measured. Exposure of isolated ILC3s to IL-23 and IL-1β induced IL-22 production (86.74 pg/mL ± 29.60), whereas purified ILC3s did not produce IL-22 in response to either bacterial species compared to no stimulation control (C: 0.92 pg/mL ± 0.19, *Rb*: 1.00 pg/mL ± 0.24, *Aj*: 1.42 pg/mL ± 0.38) (Figure 3A). In keeping with the lack of response to direct bacterial stimulation, cell surface expression of bacterial Pattern Recognition Receptors (PRRs) TLR2, TLR4, and TLR5 on colonic ILC3s *ex vivo* were low or not detected (TLR2: 0.94% ± 0.36, TLR4: 0%, TLR5: 0.60% ± 0.29) compared to Lineage+ non-lymphoid cells (TLR2: 10.23% ± 1.68, TLR4: 4.28% ± 1.22, TLR5: 7.12% ± 2.96) (Figure 3B). These data suggest that human colonic ILC3s do not produce IL-22 in direct response to the enteric bacteria tested, but require additional stimulation from accessory cells in LPMCs to induce IL-22.

### IL-23 and IL-1β Contribute to the ILC3 IL-22 Response to Gut Bacteria

**Figure 4:**
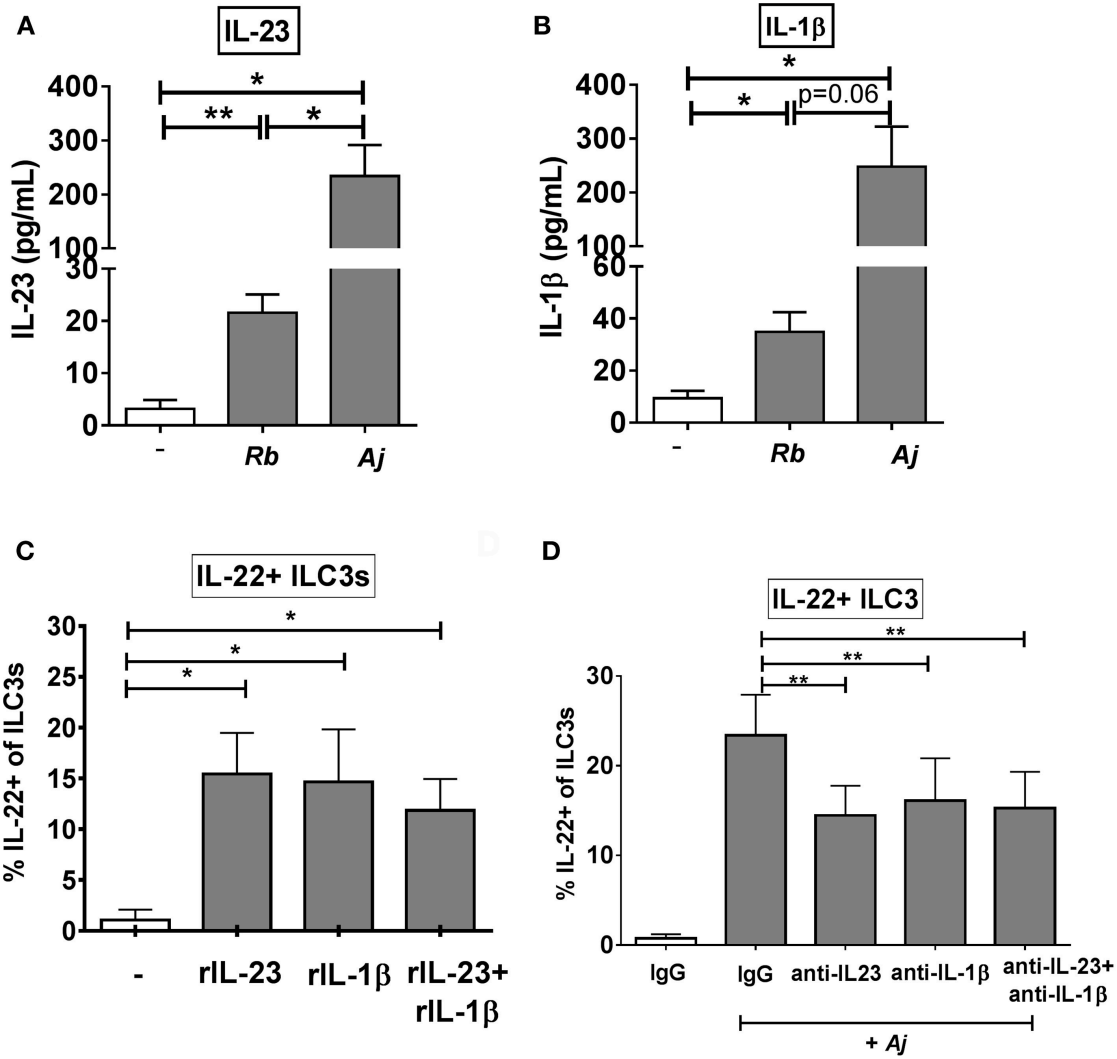
IL-23 and IL-1β modulate the ILC3 IL-22 response to enteric bacteria. **(A)** Quantification of IL-23 (pg/mL) or **(B)** IL-1β (pg/mL) in the supernatant of LPMCs exposed to *R. bromii* (Rb) or *A. junii* (Aj) or no bacteria control. *N* = 5. **(C)** Percentages of IL-22+ ILC3s after LPMC exposure to recombinant IL-23, IL-1β, or IL-23+ IL-1β (50 ng/mL) or no stimulation control. *N* = 5. **(D)** Percentages of IL-22+ ILC3s after LPMC exposure to no bacteria control or *A. junii* (Aj) in the presence of 5 ug/mL blocking antibodies targeting IL-23 and/or IL-1β or the antibody isotype control IgG. *N* = 8. Bars are mean + S.E.M. Statistical analysis performed was paired *t-*test as indicated. ^*^p < 0.05, ^**^p < 0.01.

Given that recombinant IL-23 and IL-1β stimulated production of IL-22 from purified ILC3s, the levels of secreted IL-23 and IL-1β following exposure of LPMCs to commensal Gram-positive (*R. bromii*) or to Gram-negative (*A. junii*) bacteria were next evaluated. *R. bromii* and *A. junii* significantly induced the secretion of IL-23 (*Rb*: 21.57 pg/mL ± 3.54, *Aj*: 234.4 pg/mL ± 57.23) from LPMCs (Figure 4A) above background. Both bacteria also induced significant production of IL-1β (*Rb*: 34.86 pg/mL ± 7.62, *Aj*: 247.6 pg/mL ± 74.49) from LPMCs (Figure 4B). Although both commensal bacteria stimulated significant production of these cytokines, *A. junii* induced 10.8 fold more IL-23 and 7.1 fold more IL-1β than *R. bromii* (Figures 4A,B).

To evaluate the relative contribution of IL-23 and IL-1β independently and in combination to ILC3 induction of IL-22, LPMC were exposed to recombinant IL-23 and/or IL-1β. Both recombinant cytokines individually significantly increased the percentage of IL-22+ ILC3s (C: 1.28% ± 1.22, IL-23: 17.59% ± 4.20, IL-1β: 17.41% ± 7.06) compared to the unstimulated control (Figure 4C). Although the combination of recombinant IL-23 and IL-1β significantly increased the percentage of IL-22+ ILC3s (11.07% ± 3.66) compared to unstimulated control, the combination response was not synergistic (Figure 4C).

To determine if production of IL-23 and/or IL-1β from LPMCs drives IL-22 induction in ILC3s in response to bacteria, blocking antibodies directed against IL-23, IL-1β, or both cytokines were added to LPMC cultures before exposure to the commensal *A. junii*. The frequency of IL-22 producing ILC3s in response to *A. junii* was significantly reduced by an average of 38.0% when blocking IL-23 and 38.5% when blocking IL-1β compared to stimulation with *A. junii* and the control IgG antibody (Figure 4D). The combination of blocking both IL-23 and IL-1β also significantly reduced the frequencies of IL-22 + ILC3s in response to *A. junii* by 40.1% (anti-IL-23: 14.46% ± 3.30, anti-IL-1β: 16.13% ± 4.71, anti-IL-23+ anti-IL1β: 15.28% ± 4.04), but did not lead to a synergistic reduction (Figure 4D).

Given the lack of complete abrogation of the ILC3 IL-22 response to bacteria when blocking IL-23 and IL-1β, the role of other cytokines (IL-7, IL-2) reported to promote ILC3 phenotype (57) were next investigated. Although secreted IL-7 was detected in unstimulated LPMC cultures, levels of IL-7 did not increase after LPMCs were exposed to commensal Gram-positive bacteria (*R. bromii*) or to Gram-negative bacteria (*A. junii)* (Supplemental Figure 5A). In keeping with this lack of production in presence of bacteria, blocking IL-7 alone did not reduce the frequency of IL-22+ ILC3s (Supplemental Figure 5B). Blocking IL-7 in combination with blocking IL-23 and IL-1β did not further reduce the frequency of IL-22+ ILC3s compared to blocking IL-23 and IL-1β (Supplemental Figure 5B). T cells are a major producer of IL-2 among other cytokines, however, depletion of CD3+ T cells from LPMCs did not alter the percentage of IL-22+ ILC3s generated in response to *A. junii* (Supplemental Figure 5C) and the addition of recombinant IL-2 to LPMCs did not significantly increase the percentage of IL-22+ ILC3s (data not shown). Altogether these data indicate that IL-2 and IL-7 do not have a major role in LPMCs in promoting IL-22 production by ILC3s in response to enteric bacteria in this culture system.

### Myeloid Dendritic Cells Contribute to IL-22 Production of ILC3s in Response to Bacteria by Production of IL-23 and IL-1β

**Figure 5:**
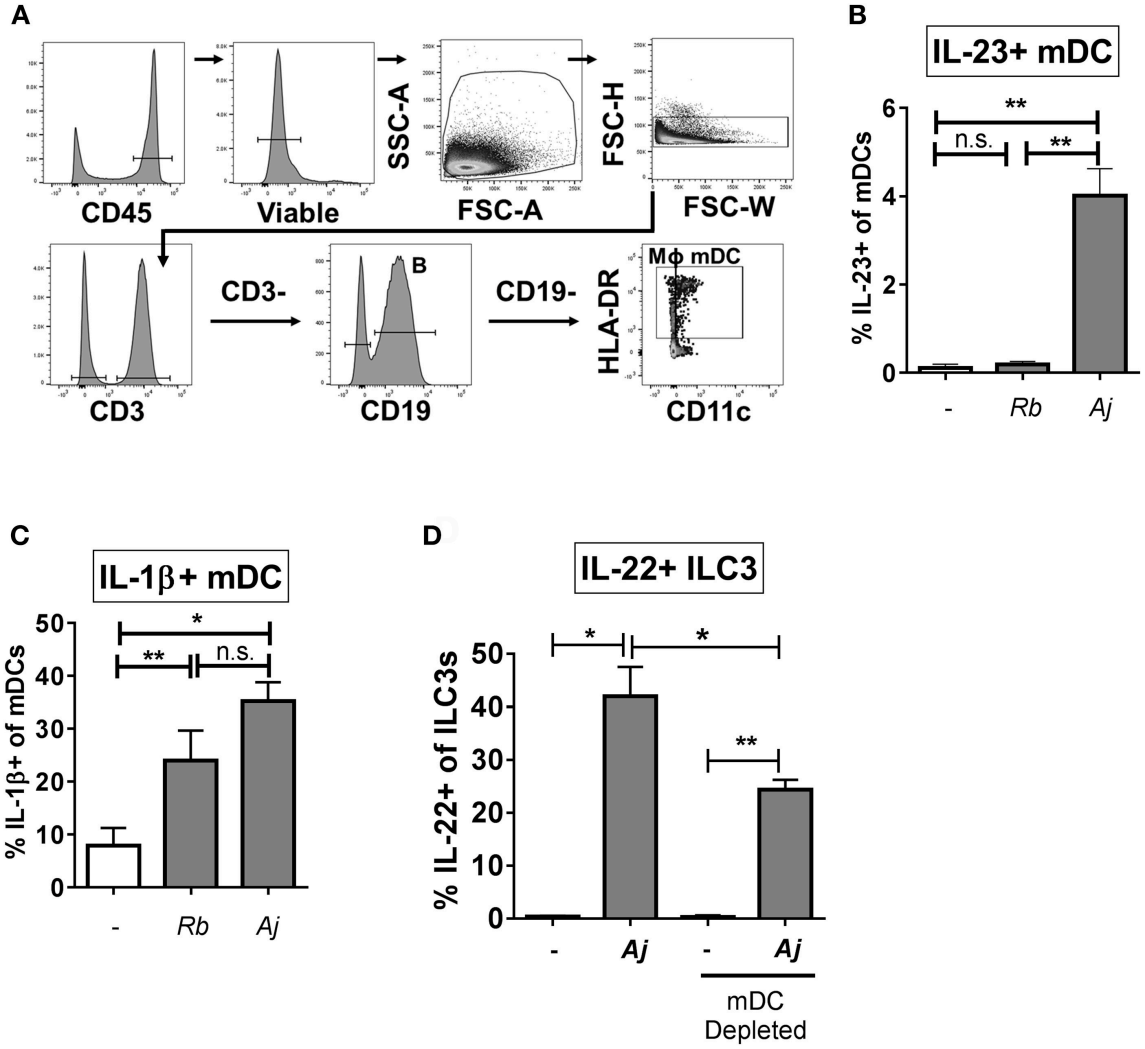
Myeloid dendritic cells producing IL-23 and IL-1β contribute to the ILC3 IL-22 response to bacteria. **(A)** Representative flow cytometry demonstrating gating strategy to identify antigen presenting cells (mDC; myeloid dendritic cells, B: B cells, MΦ; macrophages) after LPMC exposure to bacteria *in vitro*. **(B)** Percentages of IL-23+ or **(C)** IL-1β+ mDCs after LPMC exposure to *R. bromii* (Rb) or *A. junii* (Aj) or no bacteria control. *N* = 4. **(D)** Percentages of IL-22+ ILC3s after LPMC exposure to *A. junii* (Aj) or no bacteria control with CD11c mDC depletion and no depletion control. *N* = 3. Bars are mean + S.E.M. Statistical analysis performed was paired *t-*test. ^*^p < 0.05, ^**^p < 0.01. n.s., not significant.

To identify potential cellular sources of IL-23 and IL-1β in response to *A. junii* and *R. bromii*, production of IL-23 and IL-1β by mDCs (CD3- CD19- HLA-DR+ CD11c+), macrophages (defined as CD3- CD19- HLA-DR+ CD11c-) (48–50) and CD19+ B cells in LPMCs were determined by intracellular cytokine staining and flow cytometry following exposure to bacteria (Figure 5A). *A. junii*, but not *R. bromii* significantly increased the percentage of IL-23+ mDCs (Figure 5B and Supplemental Figure 6A). Although low frequencies of IL-23+ macrophages following *A. junii* stimulation were detected, this was not statistically different compared to no bacteria stimulation (Supplemental Figures 7A,B). Both *R. bromii* and *A. junii* significantly increased the percentages of IL-1β+ mDCs compared to no bacteria stimulation (Figure 5C and Supplemental Figure 6B). Exposure of LPMC to *R. bromii* or *A. junii* did not significantly induce IL-1β+ macrophages above no stimuli although IL-1β+ macrophages were detected (Supplemental Figures 7D,E). No significant increases in IL-23+ or IL-1+ B cells were observed in response to either bacteria stimuli (Supplemental Figure 7). Taken together, these observations suggested that mDCs in LPMCs were a major producer of the canonical cytokines known to drive IL-22 production by ILC3s. To verify the contribution of these cells to bacteria-induced IL-22 production by ILC3s, CD11c+ mDCs were depleted from LPMCs followed by exposure to *A. junii* and frequencies of IL-22+ ILC3s were determined. Depletion of mDCs significantly reduced the frequency of IL-22+ ILC3s in response to *A. junii* by 40.9% compared to total LPMCs (Figure 5D).

### NKp44 Ligation Contributes to the Gut ILC3 IL-22 Response to Enteric Bacteria

**Figure 6:**
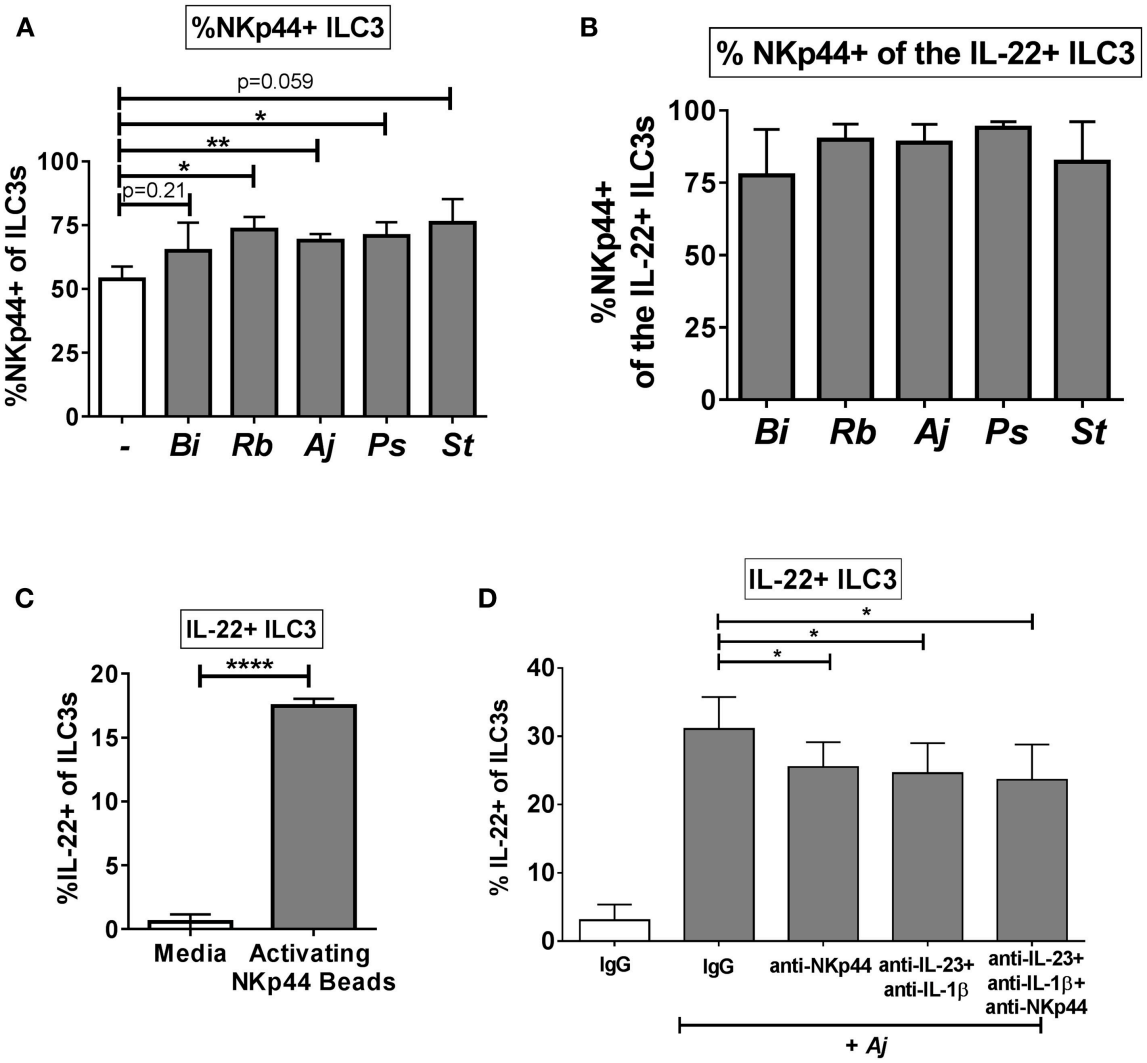
NKp44 contributes to the gut ILC3 IL-22 response to bacteria. **(A)** Percentages of NKp44 + ILC3s after LPMC exposure to enteric bacteria or no bacteria control. *N* = 5. **(B)** Percentages of NKp44+ when gating on IL-22 + ILC3s after LPMC exposure to enteric bacteria. *N* = 5. **(C)** Percentages of IL-22+ ILC3s after LPMC stimulation with NKp44 cross-linking beads. *N* = 3. **(D)** Percentages of IL-22+ ILC3s after LPMC exposure to no bacteria control or *A. junii* (Aj) in the presence of blocking antibodies targeting IL-23 and IL-1β (5 ug/mL) or NKp44 (10 ug/mL) or the antibody isotype controls IgG. *N* = 5. Bars are mean + S.E.M. Statistical analysis performed was paired *t-*test. ^*^p < 0.05, ^**^p < 0.01, ^****^p < 0.0001.

Recent work suggests a functional role for the natural cytotoxicity receptor NKp44 in driving cytokine responses of ILC3s (3), thus we sought to determine if NKp44 is critical to the IL-22 response of human colonic ILC3s to enteric bacteria. As noted previously, the majority of colon ILC3s expressed Nkp44 directly *ex vivo* (Figure 1C). Following exposure to enteric bacteria *in vitro*, a small increase in the percentage of ILC3s expressing NKp44 was noted (Figure 6A). Examination of the IL-22+ ILC3s after stimulation with bacteria revealed that the majority of IL-22-producing cells were also NKp44+ (Figure 6B). Direct ligation of NKp44 by crosslinking beads led to a significant increase in the percentage of IL-22+ ILC3s compared to the no bead control (Figure 6C). To evaluate the contribution of NKp44 to the ILC3 IL-22 response to bacteria, NKp44 was blocked in the presence of commensal *A. junii*. Compared to the IgG control, blocking NKp44 during *A. junii* stimulation resulted in a significant (IgG control: 31.08% ± 4.69, anti-NKp44: 25.52% ± 3.63) but incomplete reduction of IL-22+ ILC3s (Figure 6D). To determine if NKp44 promotion of IL-22 in ILC3s in response to bacteria was complementary or redundant with IL-23 and IL-1β, NKp44 was blocked in combination with blocking of IL-23 and IL-1β. Blocking Nkp44, IL-23, and IL-1β in combination did not further reduce the percentage of IL-22+ ILC3s generated in response to *A. junii* compared to only blocking IL-23 and IL-1β (Figure 6D).

### Bacteria-Induced IL-22 Production by ILC3s Is Enhanced in LPMCs Relative to TMCs

**Figure 7:**
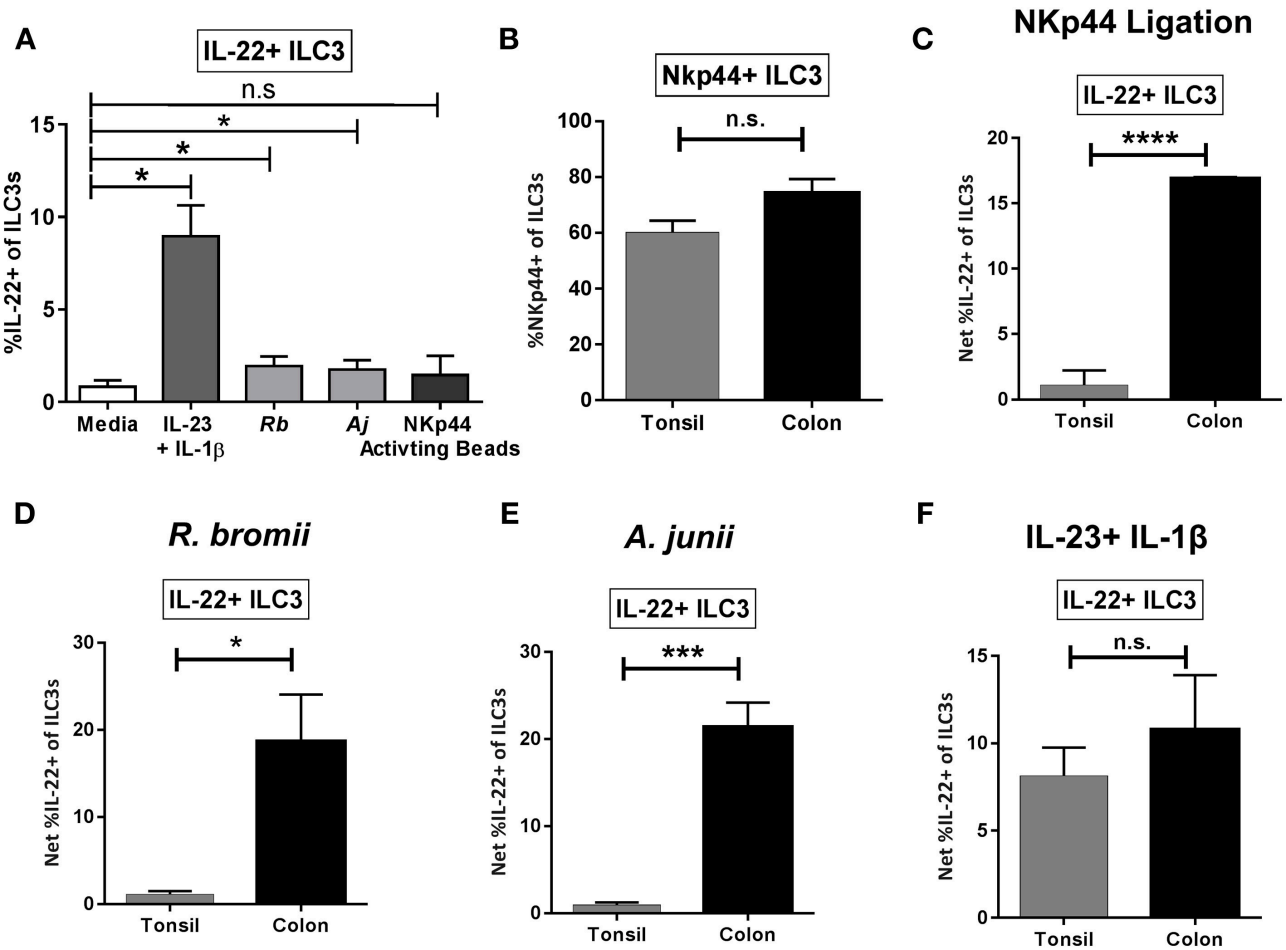
Tonsillar and gut ILC3 IL-22 responses to stimulation. **(A)** Percentages of IL-22+ ILC3s after TMC exposure *in vitro* to *R. bromii, A. junii*, NKp44 cross-linking beads, recombinant IL-23+ IL-1β (50 ng/mL) or no stimulation control. *N* = 5. Bars are mean + S.E.M. Statistical analysis performed was paired *t-*test. ^*^p < 0.05, n.s., not significant. **(B)** Percentages of ILC3s expressing NKp44 *ex vivo*. TMC: *N* = 3, LPMC: *N* = 9. **(C)** Percentages of IL-22+ ILC3s after TMC or LPMC stimulation *in vitro* with NKp44 cross-linking beads. TMC: *N* = 5, LPMC: *N* = 3. **(D)** Percentages of IL-22+ ILC3s after TMC or LPMC stimulation with *R. bromii*. TMC: *N* = 5, LPMC: *N* = 7. **(E)** Percentages of IL-22+ ILC3s after TMC or LPMC stimulation with *A. junii*. TMC: *N* = 5, LPMC: *N* = 11. **(F)** Percentages of IL-22+ ILC3s after TMC or LPMC exposure to recombinant IL-23 + IL-1β (50 ng/mL). TMC: *N* = 5, LPMC: *N* = 6. Bars are mean + S.E.M. Statistical analysis performed was unpaired *t-*test. ^*^p < 0.05, ^***^p < 0.001, ^****^p < 0.0001, n.s., not significant.

To evaluate if the ILC3 IL-22 response to bacteria is unique to the colonic environment, tonsil mononuclear cells (TMCs) were stimulated with commensal bacteria *R.bromii* and *A.junii* as well as the combination of recombinant IL-23 + IL-1β or NKp44 activating beads and induction of IL-22 by ILC3s determined by flow cytometry. The combination of IL-23+IL-1β significantly induced IL-22 production by tonsillar ILC3s as expected (Figure 7A). A small but significant increase in the percentage of IL-22+ ILC3s was observed following exposure to both commensal bacteria tested, whereas a similarly small increase in IL-22+ ILC3s following ligation of NKp44 was not statistically significant (Figure 7A). NKp44 expression on tonsillar ILC3s was characterized *ex vivo* and was not found to differ significantly from NKp44 expression on colon ILC3s (T: 60.17% ± 4.19, L: 74.21% ± 5.05) (Figure 7B). Despite the majority of the tonsillar ILC3s expressing the receptor NKp44, stimulation with NKp44 activation beads resulted in significantly lower percentages of IL-22+ ILC3s (T: 1.11% ± 1.11, L: 16.89% ± 0.12) compared to the colon (Figure 7C). Furthermore, when stimulated with bacteria, the percentage of tonsillar IL-22+ ILC3s was significantly lower in response to *R. bromii* (T: 1.11% ± 0.37, L: 18.67% ± 5.41) and *A. junii* (T: 0.93% ± 0.30, L: 21.38% ± 2.81) compared to the colon (Figures 7D,E). Although there was minimal induction of IL-22+ ILC3s in response to NKp44 ligation in tonsil cultures, the canonical cytokines, IL-23+IL-1β similarly induced ILC3 IL-22 responses (T: 8.15% ± 1.61, L: 10.79% ± 3.11) between the colon and the tonsil (Figure 7F).

## Discussion

Numerous murine studies have highlighted the importance of gut ILC3s in immunity to bacteria, but few studies have directly investigated how human lamina propria ILC3s respond to enteric bacteria, particularly commensal bacteria, and the mechanisms driving these responses. The major findings of the present study demonstrate that: (1) both Gram-positive and Gram-negative commensal and pathogenic bacteria induced similar frequencies of IL-22-producing ILC3s, (2) ILC3 production of IL-22 was not mediated through direct ILC3 recognition of bacteria, but rather mediated indirectly by mDCs, (3) IL-22 production was partly dependent on IL-23 and IL-1β, (4) and ligation of the NKp44 receptor stimulated IL-22 production. Overall, this work expands on the basic biology of human gut ILC3s and provides insight into their contribution to the innate immune response to enteric bacteria.

Similar frequencies of IL-22-producing ILC3s were induced irrespective of bacterial cell surface structure by Gram stain (i.e., Gram-positive or Gram-negative) or characterization as a human commensal or pathogenic bacteria suggesting a commonality between bacteria which drive ILC3 cytokine responses. In the context of gut ILC3 biology, this suggests that different enteric bacteria induce immune responses by LPMCs that are then “sensed” by ILC3s as the same. Using purified ILC3s, we demonstrated that induction of IL-22 was not due to direct recognition of bacteria or bacterial antigens. Presumably, direct recognition of bacteria by purified ILC3s would occur through external expression of TLRs specific to bacterial ligands. In keeping with this concept and a lack of direct induction of IL-22 by bacteria, we detected minimal expression of bacteria-associated TLRs on colonic ILC3s. This contrasts to a previous report demonstrating TLR2 expression by ILC3s in human duodenum (58) suggesting possible tissue site differences in PRR expression. Importantly, production of IL-22 by tonsillar ILC3s in response to TLR2 ligand required co-stimulation with IL-2, IL-15, or IL-23 (59).

We determined that IL-23 and IL-1β were important for bacteria-driven IL-22 production by human gut ILC3s similar to multiple murine studies highlighting IL-23-mediated regulation of IL-22 production in the context of pathogenic *C. rodentium* infection (12, 36) and responses to bacterial flagella (60, 61). IL-1β signaling through the IL-1R1 and MyD88 pathway was also shown to be critical for murine ILC3 production of IL-22 (62) and in human secondary lymphoid tissue, continuous IL-1β signaling was required to preserve the ILC capacity to produce IL-22 (63). Interestingly, in our *in vitro* model, frequencies of IL-22-producing ILC3s were not synergistically increased by the combined addition of IL-23 and IL-1β or decreased with antibody-mediated blocking of both cytokines in the context of bacteria exposure, suggesting that IL-23 and IL-1β may stimulate colonic ILC3 production of IL-22 in a redundant manner. Alternatively, ILC3s that express the IL-23R could also be the same subset that express the IL-1R indicating that only a fraction of ILC3s have to capacity to be stimulated in this manner. Taken together, these observations highlight a role for accessory cell mediated production of IL-23 and IL1β in regulating IL-22 from human gut ILC3s in response to enteric bacteria.

The importance of crosstalk between accessory cells such as mDCs and ILCs for induction of IL-22 has been implicated in a number of studies including the observation that a loss of a subset of mDCs correlated with a loss of IL-22-producing ILCs during SIV infection (25). Additionally, mouse intestinal mDC production of IL-23 in response to flagella derived from *Salmonella* was important in the stimulation of IL-22 from ILC3s (60, 61) and a need for physical contact between DCs and ILCs was necessary for the protective IL-22 response to infection with *C. rodentium* (64). We provide evidence that depletion of mDC resulted in a decrease in IL-22 production by ILC3s; however, purified colonic ILC3s responded directly to IL-23 and IL-1β, suggesting that contact dependence between human colonic ILC3s and mDCs may not be a requirement for induction of IL-22. Importantly, despite removing mDCs, complete abrogation of the bacteria-induced IL-22 response in ILC3s was not achieved, highlighting that other accessory cell types may contribute to the regulation of bacteria-specific ILC3 responses in the colon. Indeed, we detected low percentages of IL-23 and IL-1β-expressing macrophages in response to representative commensal bacteria in a subset of donors, suggesting that macrophages have the potential to contribute to the ILC3 IL-22 response to bacteria. A number of studies have implicated monocytes in driving IL-22 production. For example, soluble factors from LPS-activated human monocytes stimulated IL-22 from tonsillar ILC3s (17). In murine studies CX_3_CR1^+^ phagocytes (including mDCs and macrophages) stimulated a protective IL-22 ILC3 response during *C. rodentium* infection (65, 66) and mice deficient in CX_3_CR1^+^ phagocytes had impaired IL-22 production by ILC3s leading to increased microbial translocation and bacterial dissemination (65). Thus, exposure of multiple types of human gut antigen presenting cells to bacteria and the subsequent induction of IL-23 and IL-1β likely contributes to the production of IL-22 by ILC3s.

In Crohn's disease patients, loss of NKp44+ ILC3s from the ileum correlated with an increase in pro-inflammatory T cell subsets implying a role for NKp44+ ILC3s in mucosal regulation (67). In our study, we show that the majority of IL-22-producing ILC3s expressed the NKp44 receptor and direct ligation of the NKp44 receptor induced IL-22 from colonic ILC3s. Interestingly, the role of NKp44 in directly driving IL-22 production was unique to the gut and not observed with tonsillar ILC3s in keeping with a previous report on NKp44 ligation of tonsillar ILC3s (3). This is intriguing since we show that there are similar percentages of NKp44-expressing ILC3s *ex vivo*, as well as, similar percentages of IL-22-expressing ILC3s after *in vitro* exposure to exogenous IL-23 and IL-1β in both the tonsil and colon. In the context of bacterial stimulation, blocking NKp44 signaling partially reduced the percentage of IL-22-producing ILC3s demonstrating that NKp44 also plays a contributory role in cytokine stimulation of ILC3s. Ligands for NKp44 have been identified during pathological conditions such as tumor development or immortalized cell lines of cancerous origin, and include an isoform of the protein MLL5, proliferating cell nuclear antigen (PCNA), and platelet derived growth factor (PDGF)-DD (5, 68, 69). Further studies will be needed to determine if these, or yet to be identified ligands, can stimulate IL-22 production by ILC3s in our *in vitro* model. Interestingly, NKp44 has been shown to directly bind to bacteria (70) as well as viral hemagglutinin of influenza (71), thus, investigations will need to be undertaken to determine if enteric bacteria are an additional source of NKp44L. It is possible that other factors not identified here may contribute to the IL-22 response by ILC3s by bacteria. A recent study determined that IL-18 production by mDCs, in conjunction with IL-15, was able to induce IL-22 in tonsillar ILC3s after longer term exposure (14 days) to these cytokines (72). It is therefore possible, that similar combinations of signals could induce IL-22 from colonic ILC3s, although if there is a role for enteric bacteria in initiating this signaling cascade would need to be determined.

Reports have indicated that IFNγ production by ILC3s is possible in the context of murine models of GI bacterial infection (41, 42). Furthermore, *in vitro* human tonsillar ILC3s can be functionally plastic in terms of cytokine production including a switch from IL-22 to IFNγ production dependent on the cytokine milieu (18, 21). In this current study, exposure of LPMC to enteric bacteria did not induce a significant increase in IFNγ+ ILC3s suggesting that while our *in vitro* model has the appropriate microenvironment to drive IL-22 production from ILC3s, additional signals would be required for robust IFNγ production. It is possible that cellular movement as is seen *in vivo* could promote interactions that may be needed for IFNγ induction in response to bacteria, and the *in vitro* model used in this study may not recapitulate those interactions. It is also important to note that in our model system, broad spectrum antibiotics were present to prevent bacterial overgrowth. Thus, functional responses are likely driven by static whole bacteria and/or processed bacterial antigens. Perhaps when bacteria are able to be metabolically active and/or replicate, additional components (such as virulence factors in regards to *S. typhimurium*) would be produced leading to the subsequent production of IFNγ from ILC3s.

To the best of our knowledge, this study is the first to undertake an extensive evaluation of the mechanisms by which *in vitro* exposure to whole enteric commensal and pathogenic bacteria drive human colonic ILC3 cytokine production. Here we demonstrate that IL-22 production was driven indirectly in LPMCs, mediated in part by mDCs, and driven by multiple mechanisms including IL-23, IL-1β, and NKp44 signaling. The complexity of the gut environment was emphasized by the observation that the combination of these processes did not fully account for all of the bacteria-driven IL-22 responses. Remarkably, IL-22 production from ILC3s were induced in response to all bacteria tested, potentially highlighting an evolutionarily conserved response by ILC3s to both commensal and pathogenic bacterial antigens. IL-22 has important gut homeostatic functions and thus the production of IL-22 by ILC3s would be a critical component of the innate response to enteric pathogenic challenge. This function of ILC3s may serve as a means to repair the epithelial barrier during disease states when there is a breach in epithelium integrity to prevent the induction of further inflammation and damage by translocating microbes. Our *in vitro* observations highlight that production of IL-22 by ILC3s in response to commensal bacteria is also likely a significant component of GI tract bacterial immunity.

## Author Contributions

MC, SD, AC, EB, and CW designed the study and interpreted the work. MC, SD, and CW wrote the manuscript. MC and CP performed experiments. MM and MS provided tissue specimens. JK provided technical assistance and interpretation of the work. All authors contributed to manuscript revision, and read and approved the submitted version.

### Conflict of Interest Statement

The authors declare that the research was conducted in the absence of any commercial or financial relationships that could be construed as a potential conflict of interest.
