# Supplementary material for: Commensal and Pathogenic Bacteria Indirectly Induce IL-22 but Not IFNγ Production From Human Colonic ILC3s via Multiple Mechanisms
Source: Front Immunol. 2019 Mar 29;10:649. doi: 10.3389/fimmu.2019.00649 (PMC6450192; doi:10.3389/fimmu.2019.00649)
Supplement: Supplementary file 1 [file Data_Sheet_1.docx]

**Supplementary Material and Methods**

**Commensal and Pathogenic Bacteria Indirectly Induce IL-22 but Not IFNγ Production from Human Colonic ILC3s via Multiple Mechanisms**

Moriah J. Castleman, ^1^ Stephanie M. Dillon,^1^ Christine Purba,^1^ Andrew C. Cogswell, ^2^ Jon Kibbie, ^1^ Martin McCarter, ^3^ Mario Santiago, ^1^ Edward Barker, ^2^ Cara Wilson^1^*

^1^ Department of Medicine, Division of Infectious Disease, University of Colorado Anschutz Medical Campus, Aurora, Colorado, USA

^2^ Department of Microbial Pathogens and Immunity, Rush University Medical Center, Chicago, Illinois, USA

^3^Department of Surgery, University of Colorado Anschutz Medical Campus, Aurora, Colorado, USA

*Correspondence: Cara Wilson, cara.wilson@ucdenver.edu

**Supplemental Table 1. Bacteria Panel Investigated.** This table describes the bacteria tested in this study including characterization as Gram-positive or Gram-negative bacteria, commensal or pathogen status, relevance to HIV infection or IBD and references.

| **Bacteria Species** | **Clinical Significance** | **Reference** |
| --- | --- | --- |
| *Ruminococcus bromii* (Rb) | Gram positive commensal, decreased in relative abundance colonic mucosa in people living with HIV naïve to treatment | (Dillon et al., 2014), (Dillon et al., 2016) |
| *Acinetobacter junii*  (Aj) | Gram negative commensal, increased in relative abundance colonic mucosa in people living with HIV naïve to treatment | (Dillon et al., 2014)  (Dillon et al., 2016) |
| *Prevotella stercorea* (Ps) | Gram negative commensal, increased in relative abundance colonic mucosa in people living with HIV naïve to treatment | (Dillon et al., 2014)  (Dillon et al., 2016) |
| *Bifidobacterium infantis* (Bi) | Gram positive probiotic, therapeutic potential for restoration of microbiome | (d'Ettorre et al., 2015)  (Eom et al., 2018) |
| *Salmonella typhimurium* (St) | Gram negative pathogen, people living with HIV have increased susceptibility St bacteremia, may contribute to IBD onset | (Hung et al., 2007), (Taramasso et al., 2016), (Schultz et al., 2017) |

**Supplemental Table 2. Antibodies used for blocking assays.** This table includes information on the antibodies used in blocking assays including company and clone.

| **Company** | **Target** | **Clone** |
| --- | --- | --- |
| R& D systems | IL-23p19 | Polyclonal Goat IgG |
| R& D systems | IL-1β | Polyclonal Goat IgG |
| R& D systems | Goat IgG Control |  |
| Biolegend | NKp44 | P44-8 |
| Biolegend | Mouse IgG1 Isotype Control | MOPC-21 |
| Biolegend | IL-7 | BVD10-40F6 |
| Biolegend | Rat IgG1 Isotype Control | RTK2071 |

**Supplemental Table 3. Antibodies used for flow cytometry.** This table includes information on the antibodies used for multi-color flow cytometry including company, fluorophore used, and clone.

| **Company** | **Fluorophore** | **Target** | **Clone** |
| --- | --- | --- | --- |
| Biolegend | BV785 | CD45 | Hl30 |
| Biolegend | BV421 | CD20 | 2H7 |
| Biolegend | BV421 | CD3 | UCHT1 |
| Biolegend | BV421 | CD303 (BDCA-2) | 201A |
| Biolegend | BV421 | CD123 | 6H6 |
| Biolegend | BV421 | CD13 | WM15 |
| Biolegend | BV421 | FCεR1α | AER-37 |
| BD Bioscience | BV421 | CD11c | B-ly6 |
| Biolegend | BV421 | CD34 | 561 |
| Biolegend | BV421 | CRTH2 (CD294) | BM16 |
| Biolegend | PerCpCy5.5/APCC | CD127 | A019D5 |
| Biolegend | BV711/PE | CD117 (ckit) | 104D2 |
| Biolegend | AF700/FITC | CD56 (NCAM) | HCD56 |
| BD Bioscience | BV650 | CCR6 (CD196) | 11A9 |
| Biolegend | PE-Cy7 | NKp44 (CD336) | P44-8 |
| Biolegend | BV605 | T-bet | 4B10 |
| ebioscience | PE | RORγt | AFKJS-9 |
| ebioscience | FITC | EOMES | WD1928 |
| Ebioscience | PE-Cy7 | AHR | FF3399 |
| Biolegend | APC-Cy7 | IFN-γ | 4S.B3 |
| ebioscience | FITC | IL-22 | 22URTI |
| Biolegend | APC | IL-17A | BL168 |
| Biolegend | PE | CD11c | 3.9 |
| Tonbo | PE | CD3 | UCHT1 |
| ebioscience | PE | IL-23p19 | 23dcdp |
| Biolegend | PerCpCy5.5 | IL-12/IL-23 p40 | C11.5 |
| ebioscience | FITC | IL-1β | CRM56 |
| BD Bioscience | BV711 | CD19 | SJ25C1 |
| BD Bioscience | AF700 | CD11c | B-ly6 |
| Biolegend | PE-Dazzle | CD3 | UCHT1 |
| ebioscience | FITC | TLR2 | TL2.1 |
| Biolegend | PE | TLR4 | HTA125 |
| BD Pharmingen | AF647 | TLR5 | 624915 |

**
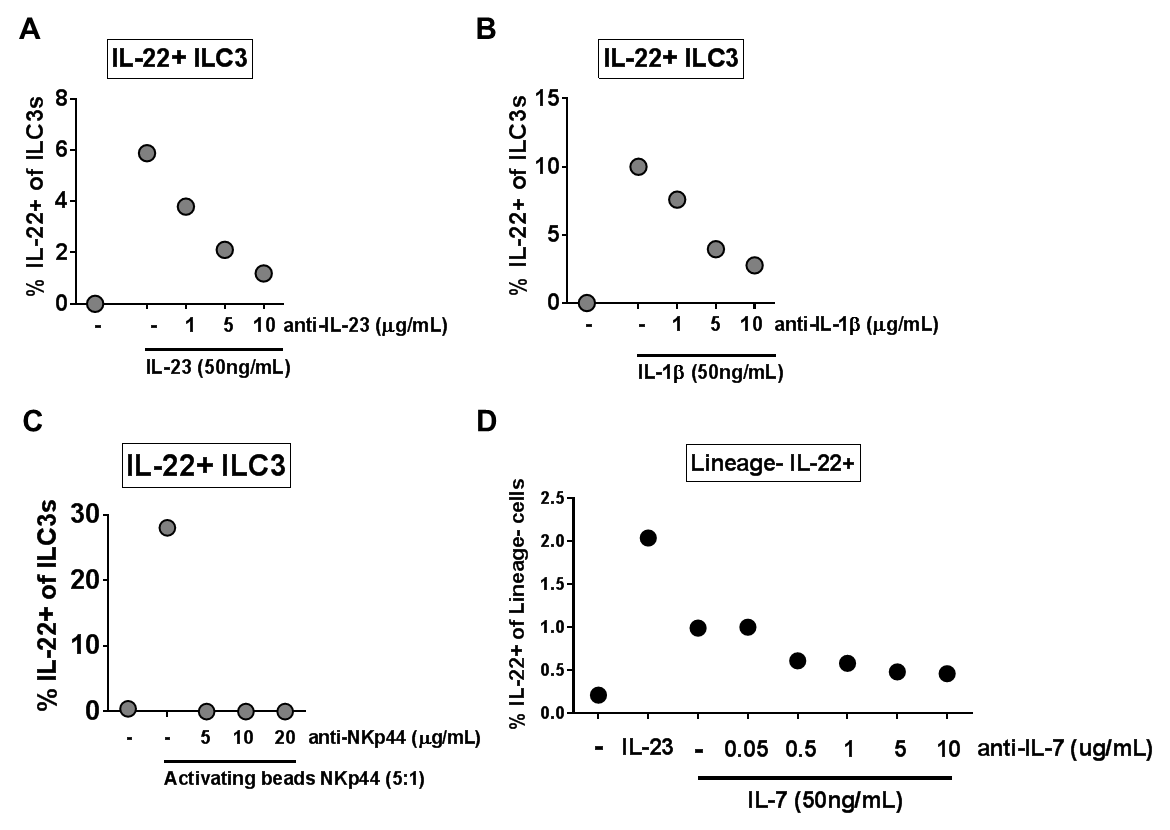
**

**Supplemental Figure 1.** **Specificity of blocking antibodies.** (A) Percentages of IL-22+ ILC3s after LPMC exposure to no stimulation control or recombinant IL-23 (50ng/mL) with increasing concentrations of blocking antibody targeting IL-23 (0ug/mL, 1ugmL, 5ug/mL, and 10ug/mL). N=1. (B) Percentages of IL-22+ ILC3s after LPMC exposure to no stimulation control or recombinant IL-1β (50ng/mL) with increasing concentrations of blocking antibody targeting IL-1β (0ug/mL, 1ugmL, 5ug/mL, and 10ug/mL). N=1. (C) Percentages of IL-22+ ILC3s after LPMC exposure to no stimulation control or NKp44 ligation beads with increasing concentrations of blocking antibody targeting NKp44 (0ug/mL, 5ugmL, 10ug/mL, and 20ug/mL). N=1. (D) Percentages of Lineage- IL-22+ cells after LPMC exposure to IL-7 (50ng/mL) with increasing concentrations of blocking antibody targeting IL-7 (0 ug/mL, 0.05ug/mL, 0.5 ug/mL, 1 ug/mL, 5ug/mL, and 10 ug/mL). Recombinant IL-23 (50ng/mL) was used as a positive control.


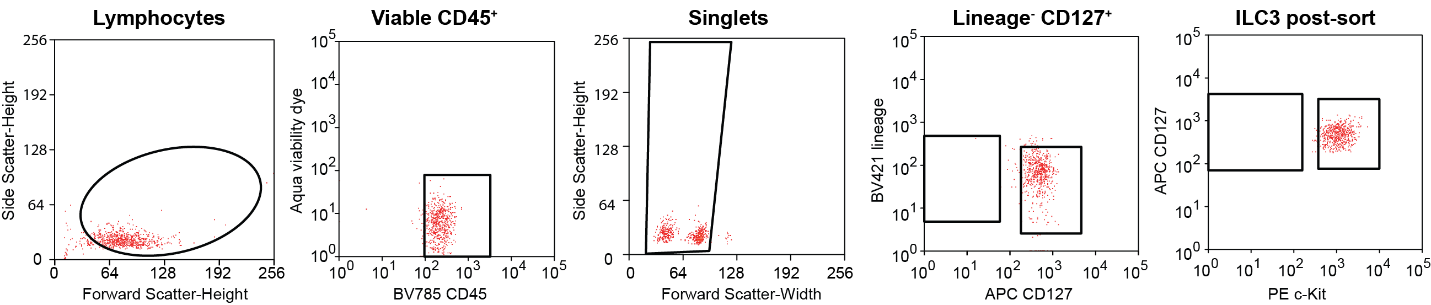


**Supplemental Figure 2.** **Purity of ILC3 populations.** Representative flow plot images of ILC3s after sorting by FACS.

**
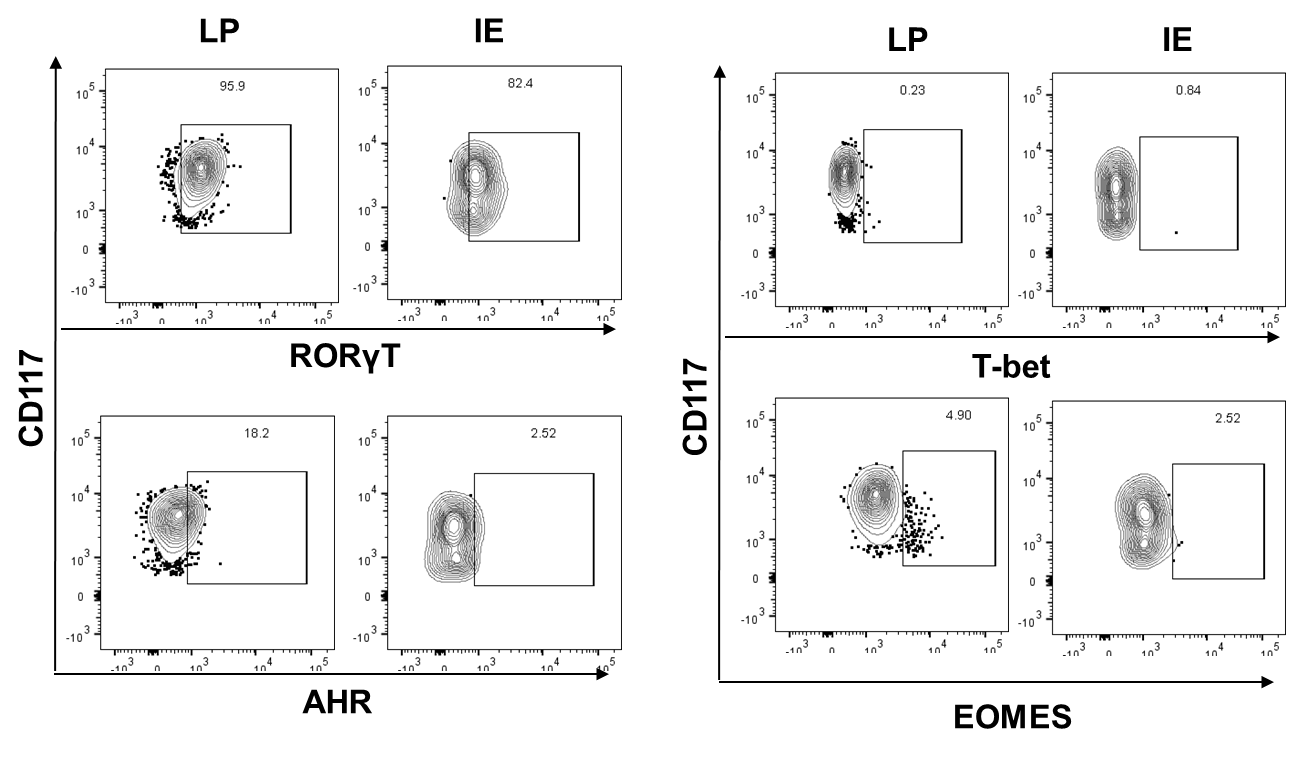
**

**Supplemental Figure 3. Flow plots for characterization of ILC3s in the lamina propria (LP) and intraepithelial (IE) layer.** Representative flow staining gated on ILC3s in both the LP and IE layer of the colon for intranuclear expression of the transcription factors RORγt, AHR, T-bet, and EOMES.


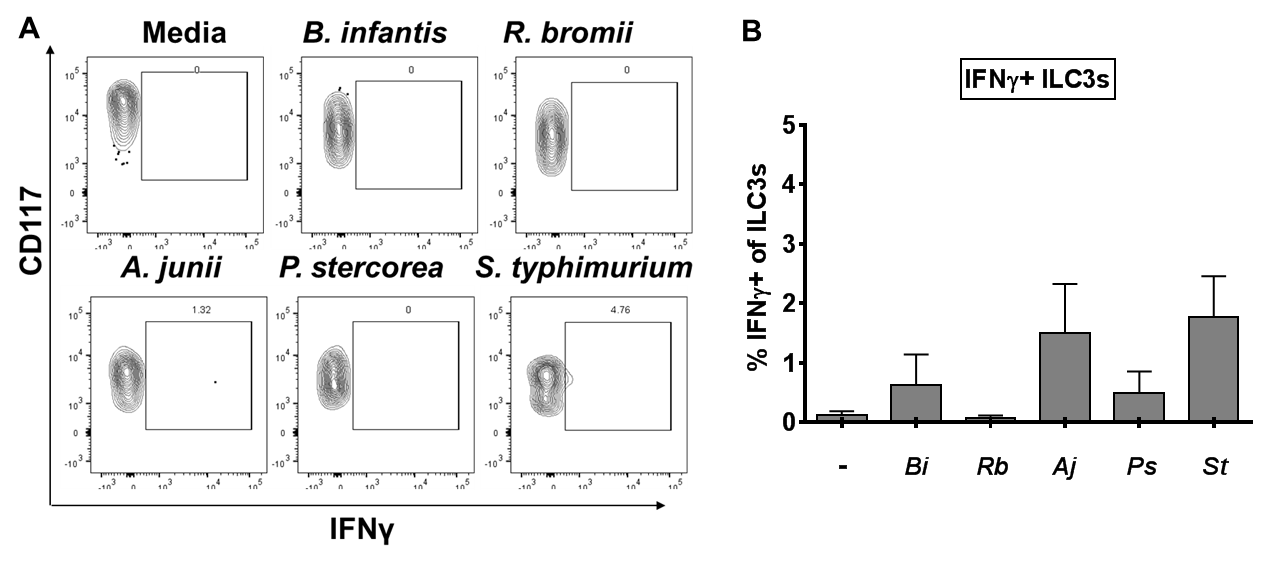


**Supplemental Figure 4. Enteric bacteria do not induce significant frequencies of IFNγ+ ILC3s compared to no bacteria control.** (A) Representative flow staining gated on ILC3s for IFNγ expression after LPMC exposure to bacteria. (B) Percentages of IFNγ+ ILC3s after LPMC exposure to bacteria. N=6. Bars are mean + S.E.M. Statistical analysis performed was paired t test between bacteria stimulated and non-bacteria stimulated control.

**
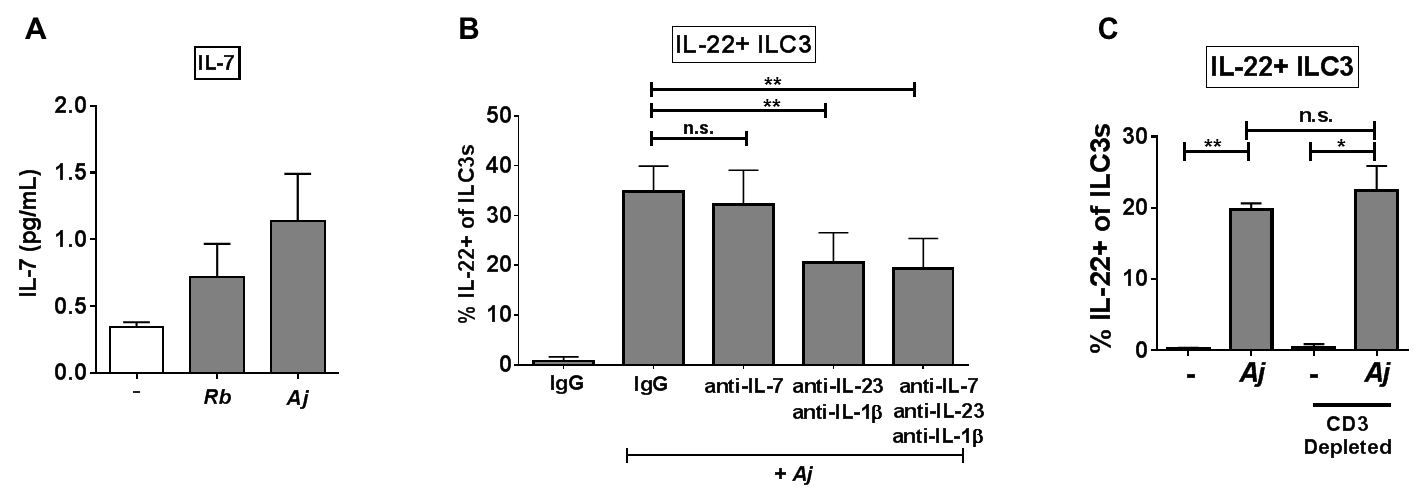
**

**Supplemental Figure 5. Minimal contribution of IL-7 or CD3+ T cells to the ILC3 IL-22 response to enteric bacteria.** (A) Quantification of IL-7 (pg/mL) in the supernatant of LPMCs exposed to *R. bromii* (Rb) or *A. junii* (Aj) or no bacteria control. N=5. (B) Percentages of IL-22+ ILC3s after LPMC exposure to no bacteria control or *A. junii* (Aj) in the presence of blocking antibodies targeting IL-23 and IL-1β (5ug/mL) or IL-7 (5ug/mL), or the combination of all three or the antibody isotype controls IgG. N=4. (C) Percentages of IL-22+ ILC3s after LPMC exposure to no bacteria control or *A. junii* (Aj) with and without depletion of CD3+ T cells. N=3. Bars are mean + S.E.M. Statistical analysis performed was paired t test as indicated. n.s.=not significant, * p<0.05, ** p<0.01.

**
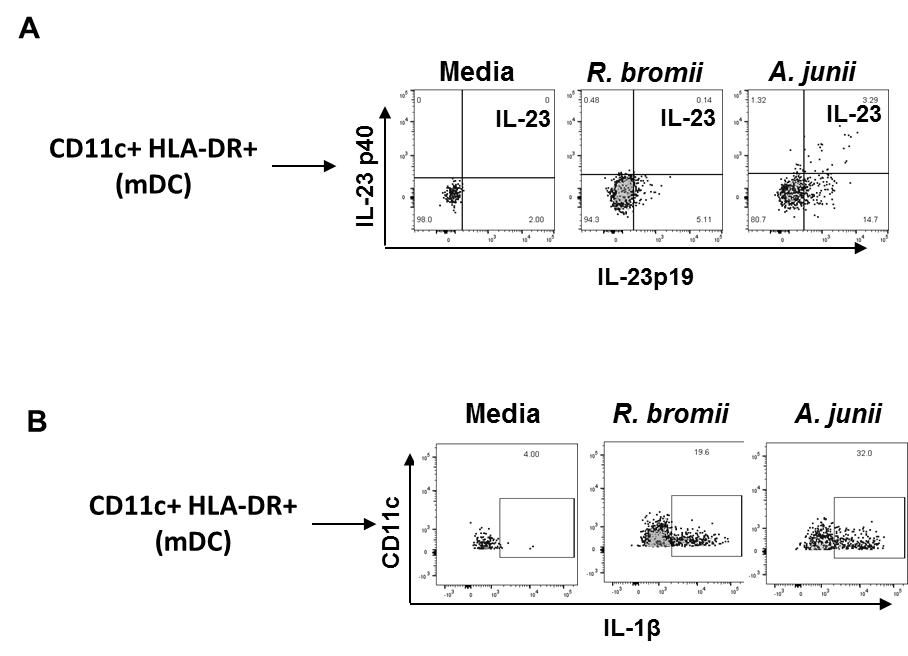
**

**Supplemental Figure 6. CD11c+ mDCs produce IL-23 and IL-1β in response to enteric bacteria.** (A) Representative flow cytometry images showing intracellular expression of IL-23 gated on viable CD45+ myeloid cells that are CD3- CD19- CD11c+ HLA-DR+ after LPMC exposure to *R. bromii* or *A. junii*. IL-23+ cells defined as positive for both IL-23/IL-12 p40 and IL-23 p19 staining. (B) Representative flow cytometry images showing intracellular expression of IL-1β gated on viable CD45+ myeloid cells that are CD3- CD19- CD11c+ HLA-DR+ after LPMC exposure to *R. bromii* or *A. junii*.

**
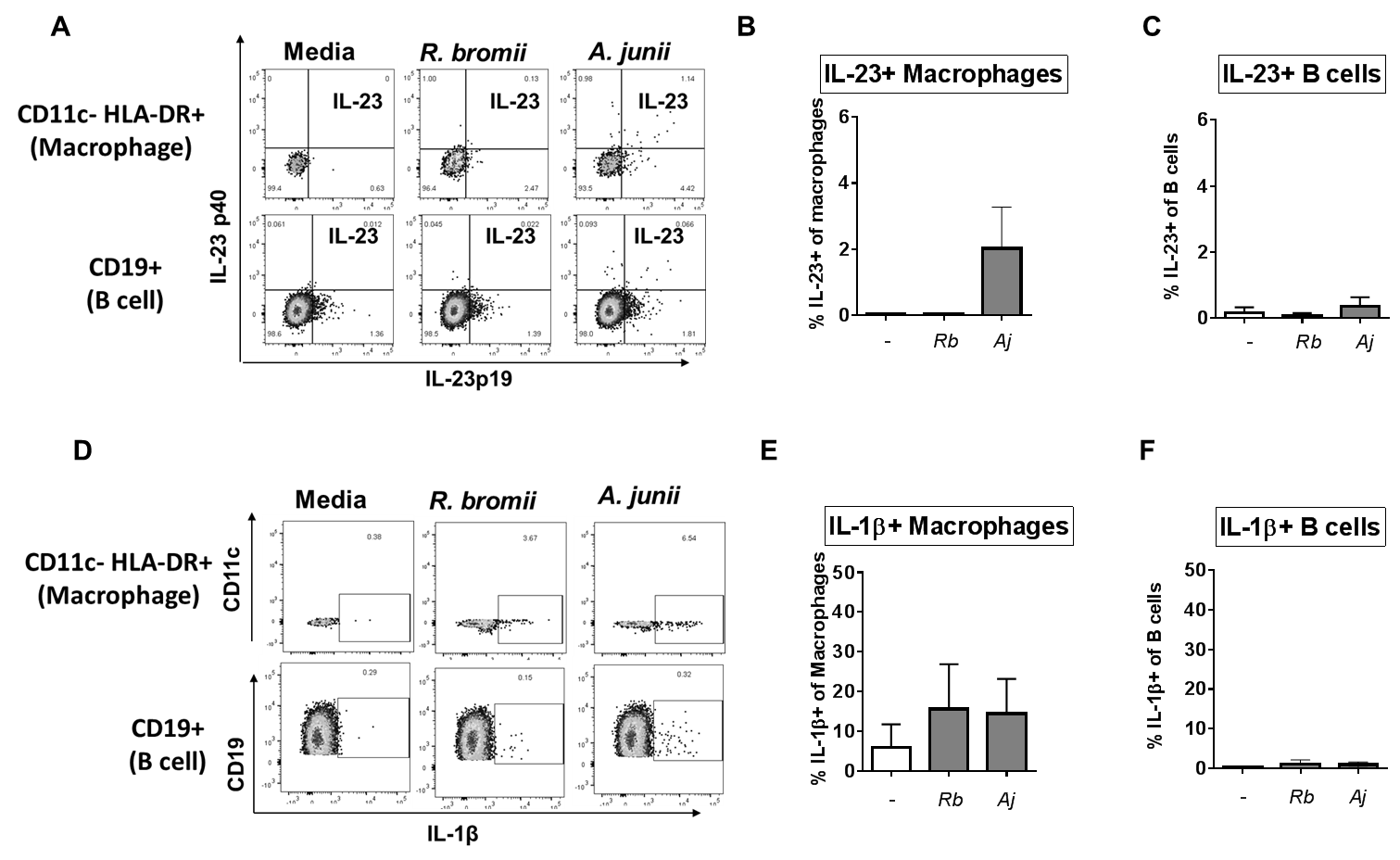
**

**Supplemental Figure 7. Minimal production of IL-23 and IL-1β by CD11c- macrophages or CD19+ B cells in response to enteric bacteria.** (A) Representative flow cytometry images showing intracellular expression of IL-23 gated on macrophages (viable CD45+ myeloid cells that are CD3- CD19- CD11c- HLA-DR+) or B cells (viable CD45+ lymphocytes that are CD3- CD19+) after LPMC exposure to *R. bromii* or *A. junii*. IL-23+ cells defined as positive for both IL-23/IL-12 p40 and IL-23 p19 staining. (B) Percentages of IL-23+ macrophages or (C) B cells after LPMC exposure to *R. bromii* (Rb) or *A. junii* (Aj). N=4. (D) Representative flow cytometry images showing intracellular expression of IL-1β gated on macrophages (viable CD45+ myeloid cells that are CD3- CD19- CD11c- HLA-DR+) or B cells (viable CD45+ lymphocytes that are CD3- CD19+) after LPMC exposure to *R. bromii* or *A. junii*. (E) Percentages of IL-1β+ macrophages or (F) B cells after LPMC exposure to *R. bromii* (Rb) or *A. junii* (Aj). N=4.

**References**

d'Ettorre, G., Ceccarelli, G., Giustini, N., Serafino, S., Calantone, N., De Girolamo, G., et al. (2015). Probiotics Reduce Inflammation in Antiretroviral Treated, HIV-Infected Individuals: Results of the "Probio-HIV" Clinical Trial. *PLoS One* 10(9)**,** e0137200. doi: 10.1371/journal.pone.0137200.

Dillon, S.M., Lee, E.J., Kotter, C.V., Austin, G.L., Dong, Z., Hecht, D.K., et al. (2014). An altered intestinal mucosal microbiome in HIV-1 infection is associated with mucosal and systemic immune activation and endotoxemia. *Mucosal Immunol* 7(4)**,** 983-994. doi: 10.1038/mi.2013.116.

Dillon, S.M., Lee, E.J., Kotter, C.V., Austin, G.L., Gianella, S., Siewe, B., et al. (2016). Gut dendritic cell activation links an altered colonic microbiome to mucosal and systemic T-cell activation in untreated HIV-1 infection. *Mucosal Immunol* 9(1)**,** 24-37. doi: 10.1038/mi.2015.33.

Eom, T., Kim, Y.S., Choi, C.H., Sadowsky, M.J., and Unno, T. (2018). Current understanding of microbiota- and dietary-therapies for treating inflammatory bowel disease. *J Microbiol* 56(3)**,** 189-198. doi: 10.1007/s12275-018-8049-8.

Hung, C.C., Hung, M.N., Hsueh, P.R., Chang, S.Y., Chen, M.Y., Hsieh, S.M., et al. (2007). Risk of recurrent nontyphoid Salmonella bacteremia in HIV-infected patients in the era of highly active antiretroviral therapy and an increasing trend of fluoroquinolone resistance. *Clin Infect Dis* 45(5)**,** e60-67. doi: 10.1086/520681.

Schultz, B.M., Paduro, C.A., Salazar, G.A., Salazar-Echegarai, F.J., Sebastian, V.P., Riedel, C.A., et al. (2017). A Potential Role of Salmonella Infection in the Onset of Inflammatory Bowel Diseases. *Front Immunol* 8**,** 191. doi: 10.3389/fimmu.2017.00191.

Taramasso, L., Tatarelli, P., and Di Biagio, A. (2016). Bloodstream infections in HIV-infected patients. *Virulence* 7(3)**,** 320-328. doi: 10.1080/21505594.2016.1158359.
